# Superhydrophobic Fatty Acid-Based Spray Coatings with Dual-Mode Antifungal Activity

**DOI:** 10.1021/acsabm.5c00596

**Published:** 2025-06-10

**Authors:** Elena Prudnikov, Hanan Abu Hamad, Iryna Polishchuk, Alexander Katsman, Ester Segal, Boaz Pokroy

**Affiliations:** † Department of Materials Science and Engineering, Technion − Israel Institute of Technology, Haifa 3200003, Israel; ‡ Faculty of Biotechnology and Food Engineering, Technion − Israel Institute of Technology, Haifa 3200003, Israel

**Keywords:** superhydrophobic, self-cleaning, coatings, fatty acids, spray coating, active components incorporation and release, antimicrobial, antifungal

## Abstract

Superhydrophobicity, a natural phenomenon commonly observed in plants and insects, imparts diverse functionalities, including self-cleaning capabilities and antibiofouling properties. Nature’s design of a superhydrophobic surface relies on a combination of surface chemistry and hierarchical roughness at micro- and nanoscales, inspiring the design of artificial superhydrophobic coatings. These multifunctional coatings offer a promising approach for combating fungal infections that are becoming increasingly prevalent due to global warming and increased resistance to conventional fungicides. Notably, among emerging superhydrophobic surfaces, those made with natural, nontoxic, and environmentally friendly compounds via facile manufacturing methods offer key advantages and support sustainable engineering practices. In this study, we developed easy-to-apply, sprayable bimodal superhydrophobic coatings. The antifungal activity of these coatings, based on long-chain fatty acids, can be further enhanced by incorporating medium-chain fatty acids, as demonstrated against the model phytopathogen . Specifically, we investigate the effect of incorporating sorbic or caprylic medium-chain fatty acids at various concentrations on the structure, physical properties, stability, and applicability of stearic acid-based coatings. Our results show that, depending on the composition, the antifungal activity of the coatings can be tuned, ranging from complete passive antibiofouling to dominant fungicidal action against . Enabled by the synergistic effect of the hierarchical superhydrophobic structure and the incorporation of potent medium-chain fatty acids, these coatings offer a sustainable solution for surface protection against fungal infections and represent a promising alternative to conventional antifungal strategies.

## Graphical abstract

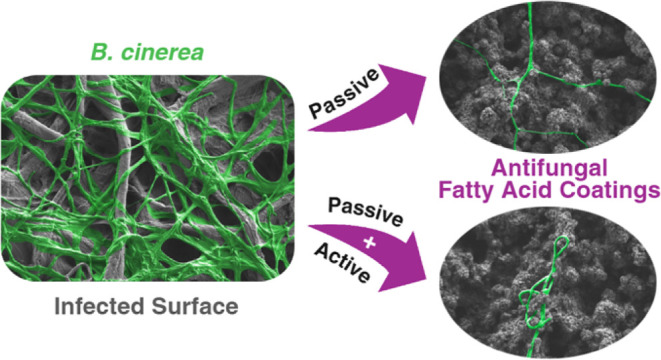

## Introduction

Superhydrophobicity is a phenomenon widely found in nature, especially among plants and insects.^1–4^ Organisms and plants employ this strategy to achieve diverse functionalities, including self-cleaning capabilities and improved flight performance.^5,6^ Among them, the Lotus plant has been studied over the last decades for its unique wetting properties, serving as a foundation for research into superhydrophobic natural surfaces and the development of artificial man-made analogs.^1,5,7,8^ Beyond water repellency, superhydrophobic surfaces can impart additional functionalities such as thermal insulation, corrosion prevention, antifouling properties, anti-icing, antifogging, and more.^1,2,9–14^ This unique behavior arises from the interplay between two key factors: surface chemistry and surface roughness.^1–3^ Hydrophobic molecules on the surface inhibit strong physical bonds with water, which is an essential but insufficient condition for superhydrophobicity.^1,2,15,16^ The synergy between the appropriate chemistry and a combination of micro- and nanoscale roughness yields contact angles exceeding 150°, characteristic of superhydrophobicity.^1,2,15–17^ The Lotus plant exemplifies this synergy, coupling high water repellency with a self-cleaning effect driven by low contact angle hysteresis (CAH <10°), a key requirement for artificial superhydrophobic surfaces.

Various methods are employed to create superhydrophobic surfaces or other self-cleaning strategies, including deposition techniques, lithography and templating methods, sol–gel processes, plasma and chemical etching, electrochemical processes, 3D printing, SLIPS, and others.^18–24^ Our group has developed a bioinspired method to form crystalline superhydrophobic surfaces by self-assembling paraffin waxes deposited through thermal deposition, mimicking the wax crystals covering lotus leaves.^25,26^ The properties of these surfaces can be accurately tuned and demonstrate excellent antibiofouling behavior.^27–30^ Building on this, we explored fatty acids as an alternative material for creating superhydrophobic surfaces through molecular self-assembly.^31^ Fatty acids offer several advantages, including low cost, natural abundance in biological systems (including the human body), and well-documented antimicrobial properties, although the exact mechanism behind these properties remains not fully understood.^32,33^ Recently, fatty acids have gained significant attention as a formulation component for various superhydrophobic surfaces, including fabrics and edible coatings.^34–40^ Our previous study demonstrated the feasibility of using a spray coating method to form superhydrophobic and self-cleaning surfaces with hierarchical roughness using a solvent-based formulation comprising long chain saturated fatty acids (16–20 carbons).^31^ We showed that these fatty acid coatings provided protection against and due to their unique surface morphology and roughness, despite the lack of intrinsic antibacterial properties in the powdered fatty acids.^31^ Shorter fatty acids (≤10 carbons), unlike long-chain fatty acids (≥12 carbons) are known for their more potent inherent antimicrobial activity.^32,33^ Likewise, unsaturation of the fatty acid chain increases the antimicrobial potency when the same chain length is considered.^32,33^ However, the physical properties of fatty acids, determined by their molecular length and degree of saturation, significantly impact their suitability for forming superhydrophobic coatings.^32,33,41^ Shorter-chain fatty acids tend to be more hydrophilic and often exist in liquid form, rendering them unsuitable as standalone components for superhydrophobic coatings.

In this study, we expand the previously introduced concept of superhydrophobic fatty acid coatings by developing coatings that incorporate multiple fatty acids for enhanced antimicrobial activity using a facile spray-coating method.^42^

Two medium-chain fatty acids (MCFAs), sorbic and caprylic acids, were selected as potent additives to the long-chain stearic acid–based coating. Sorbic acid (C_6_H_8_O_2_) is a solid polyunsaturated fatty acid well-known for its antimicrobial properties and is widely used as a food preservative.^43,44^ Caprylic acid (C_8_H_16_O_2_), a liquid saturated fatty acid, which is an effective antimicrobial agent against both bacteria and fungi.^45–47^ This study not only examines the structure, morphology, and crystallography of these bimodal surfaces and the impact of medium-chain fatty acids on the performance of long-chain fatty acid coatings, but also explores the antifungal properties of both single-component and newly developed multicomponent fatty acid coatings.

With global warming, the detrimental impact of fungi on humans is on the rise,^48^ inducing the emergence of new fungal pathogens, their spread, and antifungal resistance.^49^ Fungi directly threaten human health, causing severe invasive infections that are estimated to result in over 2.5 million deaths globally.^50^ They also account for indirect economic effects.^51^ Yet, despite these staggering figures, there are only a handful of reports that explore the use of bioinspired patterned surfaces to mitigate fungal pathogens on various surfaces.^52^

In this study, (), commonly known as gray mold, was selected as a model fungus. is a prevalent necrotrophic fungus that attacks plants through various mechanisms, making it difficult to control.^53,54^ Mitigating fungal infections, including those caused by , is particularly challenging due to its ability to develop resistance to commonly used fungicides.^54^ Additionally, conventional fungicides often pose risks to both human health and the environment, which leads to regulatory restrictions.^54^ Therefore, there is an urgent need for effective and sustainable disease management strategies to reduce the reliance on synthetic fungicides.^54–56^ Fatty acids emerge as promising antifungal agents as they are less likely to induce resistance development and are environmentally friendly.^46,47^

Herein, we correlate the compositional, structural, and wetting properties of single- and multicomponent fatty acid superhydrophobic coatings with their antifungal activity against , demonstrating their ability to resist fungal infections.

## Experimental Section

### Materials

Stearic acid (97%, Merck, Germany), sorbic acid (99%, Thermo Scientific, USA), and caprylic acid (99%, Acros Organics, USA) were used for coating formation. Diethyl ether (stabilized with BHT, Bio-Lab, Israel) was used as a solvent.

### Coating Formation

A stearic acid solution at a concentration of 20 mg/mL was prepared by adding stearic acid to diethyl ether and stirring until complete dissolution was achieved. Then, the required amount of sorbic acid or caprylic acid, according to the desired coating composition, was added to the stearic acid solution and manually mixed to achieve complete dissolution (details in Table S1). The solutions were applied onto microscope glass slides using a commercially available spray gun (Airbrush BRIXO Tank Top Airforce, 600 cc, nozzle size 1.4 μm) connected to compressed air at a pressure of 6 bar. The spraying procedure induces the evaporation of the diethyl ether, followed by the recrystallization of fatty acids to form the coating. A constant volume of 50 mL was sprayed onto a standard glass slide (7.5 × 2.5 cm^2^) to fabricate the coatings. Samples were characterized and stored in separate Petri dishes at ambient conditions.

### Characterization

#### Electron Microscopy

The coating̀s morphology was studied using a high-resolution scanning electron microscope (HR-SEM)—Zeiss Ultra Plus FEG-SEM 1 kV voltage was applied during the imaging. Prior to the measurement, the samples were coated by using a carbon coater.

#### Surface Roughness

Surface roughness measurements and surface visualization were performed by using a dynamic confocal microscope (Leica DCM3D or Sensofar Sneox). The confocal microscope data were processed using SensoMap Turbo 5.1.1.5450 or SensoView 2.2.2 software, respectively, to obtain surface visualizations and roughness parameters.

#### Wetting Properties

The wetting properties, contact angle, and contact angle hysteresis were measured using an Attension Theta Lite tensiometer and high-purity water. A volume of 7 μL was used for static contact angle measurements.

#### Structural Analysis

Structural characterization of the coatings was performed by X-ray diffraction (XRD) using a Rigaku SmartLab 9 kW diffractometer in parallel beam theta-2theta measurement mode. High-resolution powder X-ray diffraction (HR-PXRD) was performed on beamline ID22 of the European Synchrotron Radiation Facility (ESRF) in Grenoble, France. The coatings were detached from the substrate (mechanically scraped off) to perform these measurements, and the resulting powder was measured inside a spinning capillary.

### Coatings on a Flexible Porous Substrate (Cellulose Filter Paper)

The coating on the filter papers was prepared following the previously described procedure, with 100 mL of the solution sprayed over a standard 90 mm Petri dish containing seven filter papers fixed in place (Whatman, grade 1, 25 mm, China). The properties of the coatings deposited on the paper substrates were characterized as described above.

To assess their long-term superhydrophobic performance, a 7 μL water droplet, colored with methyl orange for easier observation, was placed on the coated filter papers. The samples were then covered with a Petri dish to minimize water evaporation during observation.

To study the performance of the coatings under challenging water exposure, the coated filter papers were placed coating-side-up on the water surface, allowing the uncoated side of the filter paper to readily absorb water. A water droplet (7 μL) stained with methyl orange was placed on the coated moist substrate, and the wetting properties of the coatings were monitored over time to evaluate their durability and functionality under these conditions.

### MCFA Leaching

To study the leaching of medium-chain fatty acids (MCFAs), specifically caprylic and sorbic acids, from the coatings, similarly sized samples were used in each experimental set (square samples of approximately 1 × 1 cm^2^ or round samples with an 18 mm diameter). Freshly coated samples were immersed in 5 mL of double-distilled water for varying durations, ranging from 15 min to 24 h. After immersion, the corresponding residual aqueous medium was collected and analyzed, while the coatings’ morphology and properties were characterized. In another set of experiments, samples were immersed in double-distilled water for up to 11 days, and the residual aqueous medium was analyzed in a similar manner.

#### Absorbance Measurements

Aliquots of 200 μL were spectrophotometrically analyzed using a microplate reader (Varioskan Flash, Thermo Fisher Scientific, USA or Multiskan GO, Thermo Fisher Scientific, USA). The measurements were performed in the wavelength range of 200–400 nm.

#### Thermogravimetric Analysis (TGA)

Thermogravimetric analysis (TGA Q5000, TA Instruments, USA) was utilized to quantify the release of caprylic acid from the coatings under accelerated conditions. The coating samples were heated to 45 °C in an air atmosphere, and the weight loss was monitored continuously for 10 h. To assess the residual content of caprylic acid in the coating, the same sample was subsequently analyzed in a dynamic high-resolution mode (resolution number: 5; sensitivity value: 1). In this experiment, the sample was heated at a rate of 5 °C min^–1^ up to 350 °C in a nitrogen atmosphere. TGA results were analyzed by Universal Analysis 200 version 4.5A build 4.5.0.5 software.

### Antifungal Studies

#### Fungal Strain and Materials

, collected from (isolate B05.10), was kindly supplied by Prof. Amir Sharon (Tel Aviv University, Israel). Potato dextrose agar (PDA) was supplied by Sigma-Aldrich (Israel). FUN-1 cell stain was obtained from Thermo Fisher Scientific Inc. (USA). Milli-Q water (18.2 MΩ·cm) was used in all experiments.

#### Antifungal Assays

Antifungal studies were conducted on PDA at 24 °C. Fungal spores were harvested and dispersed in 0.9% saline, and the concentration of the resulting spore suspension was adjusted to 10^4^ spores mL^–1^ using a hemocytometer. 100 μL of this suspension was spread on PDA plates using glass beads. 50 mg of the studied fatty acid (either as powder or liquid) was placed at the center of the dish and incubated at 24 °C for 7 days. In the case of caprylic acid (liquid MCFA), a round hole in the agar was created with a puncher, and a volume of 55 μL (equivalent to 50 mg) of the acid was introduced. The mycelial growth was visually observed, and the diameter of the inhibited growth area was measured by using a digital caliper.

The antifungal properties of the coatings were tested using cellulose filter papers as a substrate. Briefly, the filter papers were sterilized by using UV radiation prior to the deposition of fatty acid formulations. The coated filter papers were placed on PDA plates with their coated sides facing up. Mycelial agar plugs (5 mm diameter and 5 mm thickness) containing actively growing fungi were obtained from the edge of a 7-day-old culture. These plugs were placed on top of the coated samples, and the plates were incubated at 24 °C for 72 h in the dark. The samples were visually analyzed, and the visible mycelial growth area was measured using a digital caliper. Experiments were done in *n* = 6, and statistical significance was evaluated using Student’s two-tail *t* test, *p < 0.05 is considered statistically significant. Optical microscopy (Olympus BX51) was employed to visualize the fungal mycelium and its extent of growth on the samples. Additionally, the viability of was investigated by FUN-1 staining to differentiate between metabolically active and inactive or dead fungal cells.^57–59^ Following 72 h of incubation, the samples were detached from the agar plate and transferred onto microscope slides. A 10 mM stock solution of FUN-1 was diluted to a working concentration of 10 μM in distilled water, and 30 μL of this solution was applied to the filter paper. The samples were visualized using a Confocal Laser Scanning Microscope (CLSM) LSM 880 – Upright Confocal Microscope equipped with a multiline argon laser set to an excitation wavelength of 488 nm. Z-stack images were captured along the *z*-axis of the samples, with emissions detected at 521 nm for the green channel and 630 nm for the red channel, facilitating the differentiation of viable from nonviable cells based on their distinct fluorescence profiles. Note that FUN-1 is a membrane-permeable fluorescent dye, which is extensively used in studies of yeast and other fungi to monitor cell viability.^60^ While the dye is internalized by both living and dead cells with intact plasma membranes, resulting in a diffuse yellow-green fluorescence within the cytoplasm, only in metabolically active cells is green fluorescence converted into orange-red cylindrical intracellular structures (CIVS), providing an indicator of cell viability.

Fungal morphology of the samples was studied by scanning electron microscopy (Zeiss Ultra Plus FEG-SEM). The samples were fixed by incubation overnight in 2% glutaraldehyde solution, followed by washing and vacuum drying.

## Results and Discussion

### Preparation and Characterization of the Coatings

To examine the effect of the presence of MCFAs in the coating̀s composition, stearic acid solutions containing various concentrations of sorbic or caprylic acids (from 10 to 40% of the total weight of fatty acids in the solution; see details in Table S1) were spray-deposited onto glass slides. The samples were then characterized and monitored over a 2-month period while stored in separate Petri dishes at ambient conditions.

Figure 1a–h illustrates the impact of caprylic acid addition on the final morphology and properties of the coating. The gradual increase in caprylic acid content results in the formation of smoother spheres, lacking the well-defined plate-like crystal morphology observed in pure stearic acid coating (Figure 1a–e). The water contact angle decreases from 163° in the pure stearic acid coating to 86° when the coating contains 40% caprylic acid. Additionally, CAH increases from 3° to an unmeasurable level as caprylic acid is added, resulting in the loss of superhydrophobicity and self-cleaning ability even at just 10% caprylic acid (Figure 1f,g). The inclusion of caprylic acid, with its shorter aliphatic chain and lower hydrophobicity compared to stearic acid, accounts for these changes. Moreover, these coatings demonstrate roughness values (root-mean-square, denoted as Sq) that progressively increase from 6.8 to 21.0 μm with the addition of caprylic acid (Figure 1h). This increase indicates the presence of more pronounced crystal assembly during the spraying process, as evidenced by the corresponding HR-SEM images (Figure 1a–e). Roughness parameters related to the horizontal surface dimensions,^61^ namely the developed area ratio (Sdr) and the density of peaks (Spd), exhibit a similar trend, with a slight deviation observed in the 10% caprylic acid sample (see details in Figure S1a,b). This indicates the formation of a coarser and rougher surface with a higher caprylic acid content, consistent with the observations from the HR-SEM images (Figure 1a–e).

**Figure 1:**
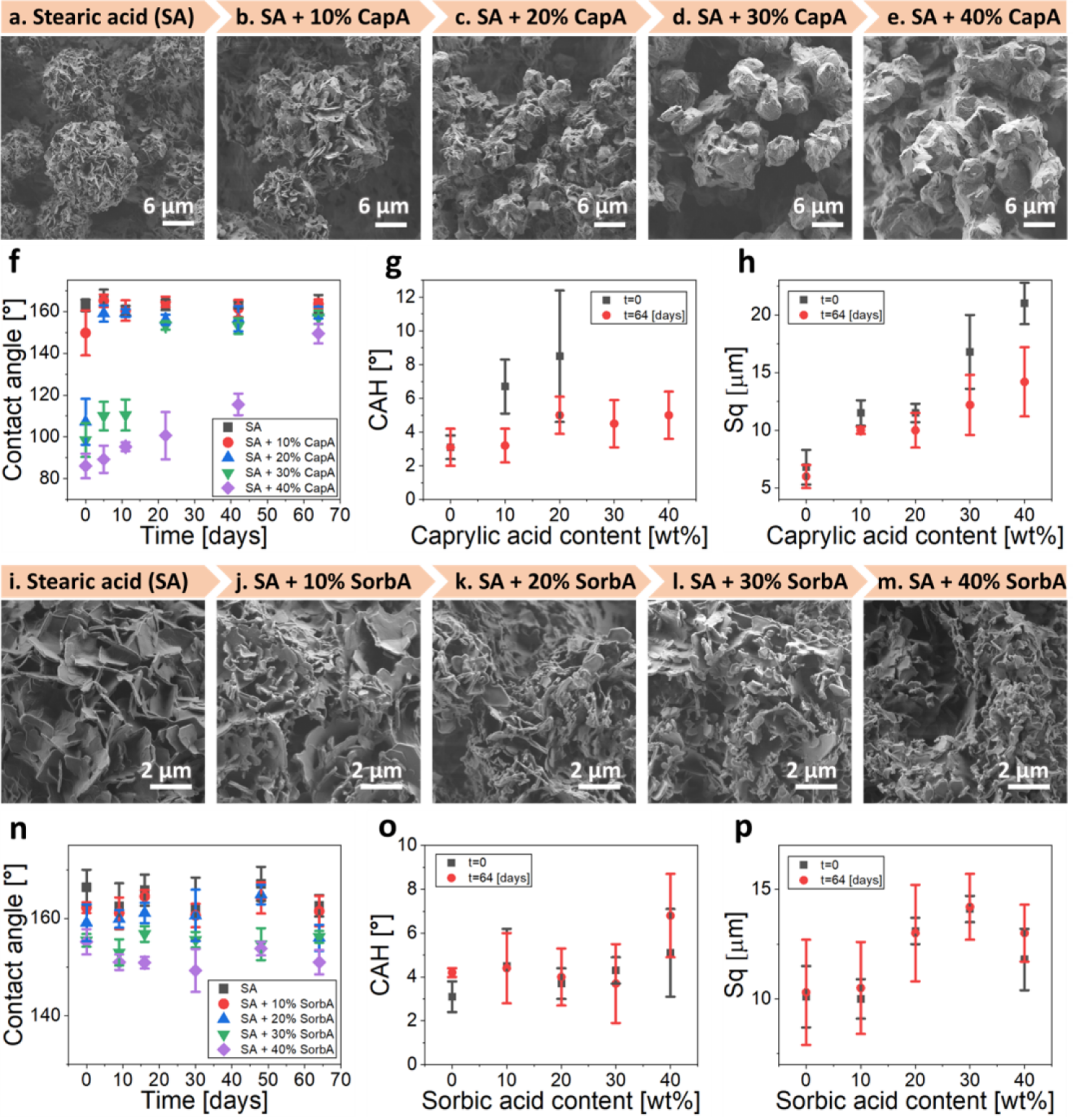
(a–h) Characterization of the coatings with caprylic acid. (a–e) HR-SEM images of sprayed stearic acid coating with various amounts of caprylic acid (CapA) – 0%, 10%, 20%, 30%, and 40%, respectively. (f) Water contact angle evolution over time across different coatings. (g) Contact angle hysteresis of the different coatings at *t* = 0 and *t* = 64 days. (h) Roughness of the different coatings at *t* = 0 and *t* = 64 days. (i–p) Characterization of the coatings with sorbic acid. (i–m) HR-SEM images of sprayed stearic acid coating with various amounts of sorbic acid (SorbA) – 0%, 10%, 20%, 30%, and 40%, respectively. (n) Water contact angle evolution over time across different coatings. (o) Contact angle hysteresis of the different coatings at *t* = 0 and *t* = 64 days. (p) Roughness of the different coatings at *t* = 0 and *t* = 64 days.

These results suggest that following the initial formation of stearic acid nuclei, the local concentration of caprylic acid around the crystals increases, probably resulting in its adsorption onto the stearic acid crystals. As the solvent evaporates during spraying, the concentration of stearic acid crystals surrounded by caprylic acid in each droplet increases. It is important to note that caprylic acid is liquid at RT, giving it an oily appearance, and does not crystallize on its own. Finally, stearic acid crystals assemble via adsorbed caprylic acid liquid layers. This process leads to stacking of the formed crystals into smooth spheres and their coalescence into clusters (Figure 1h,a–e).

Interestingly, the CA of all coatings containing caprylic acid increased over a period of 2 months. In particular, coatings with up to 30% caprylic acid eventually demonstrate values of CA higher than 150°. The higher the caprylic acid content in the sample, the longer it takes to reach a CA of 150° (Figure 1f). A coating with 10% caprylic acid exhibited a CA characteristic of superhydrophobic surfaces in just 5 days, whereas 42 days were required in the case of a coating with 30% caprylic acid to showcase similar behavior. This gradual increase in CA over time, without accompanying changes in the morphology (Figure S2a,e), likely suggests the slow evaporation and release of caprylic acid from the outer surface of the coating, allowing the recovery of the superhydrophobic properties typical of pure stearic acid coating. This assumption is further evaluated and discussed (details in Figure 4).

The addition of sorbic acid to the coating’s composition had a limited effect on its properties. The spraying process activates solvent evaporation, followed by the crystallization of both sorbic acid and stearic acid, which are crystalline solids at RT. The coating morphology is slightly altered by the addition of sorbic acid (Figure 1i–m). The inclusion of sorbic acid in the formulation results in the formation of smoother, less-defined crystals, which are distributed between the primary plate-like crystals that form the spherical crystal assemblies (Figure 1i vs j–m and lower magnification images with a larger field of view are depicted in Figure S3). The effect becomes more pronounced as the amount of sorbic acid increases. Interestingly, the contact angle of the resulting coating only slightly decreases with the higher sorbic acid content, despite its relatively high solubility in water. Moreover, the contact angle remains constant over time (Figure 1n). In contrast to the coatings with caprylic acid, all the tested compositions with sorbic acid (up to 40% w/w) showed water contact angles higher than 150° and CAH lower than 6° at *t* = 0, indicating the formation of superhydrophobic surfaces demonstrating self-cleaning properties (Figure 1n,o). Only minor deviations from the initial values, within the margin of error, were observed over time. The coating’s roughness (Sq) and horizontal surface parameters (Sdr and Spd) are less impacted by the presence of sorbic acid compared to coatings containing caprylic acid (Figures 1h,p and S1d,e). No significant changes in the morphology of the various samples were observed after 2 months of storage (Figure S2f–j).

To determine whether the presence of caprylic or sorbic acids alters the coating’s structure, we conducted X-ray diffraction in θ-2θ mode, using a wavelength of 1.54 Å.

The collected X-ray diffraction patterns are listed in Figure 2. Analysis of the pure stearic acid coating (Figure 2a,b, black line) reveals the presence of two polymorphs of stearic acid: the C-form, which also exists in the original powdered material (Figure S4), and the E-form, which emerges as a result of the spray deposition process. Both structures are bilayers composed of two molecules, with the carbon chains in an all-trans conformation.^62^ The C-form is the high-temperature phase, but once formed, it remains metastable at RT for at least 2 years.^62^ Given that crystallization occurs during the spray process at relatively low temperatures, the dominant polymorph formed is the E-form rather than the C-form. A preferred orientation of the (00l) planes for both C and E forms is clearly observed in the pure stearic acid diffraction pattern compared to powder diffraction or theoretical data for the different polymorphs (Figure S4, CIF: C-form: 1263279, E-form: 1263283). Notably, the (003)_E_ plane associated with the E-form exhibits a higher intensity than the corresponding plane for the C-form. This difference suggests that the E-form is the predominant phase in the spray-deposited coating (Figure 2, black line), as the intensities of these peaks would be similar in a randomly oriented powder sample.

**Figure 2:**
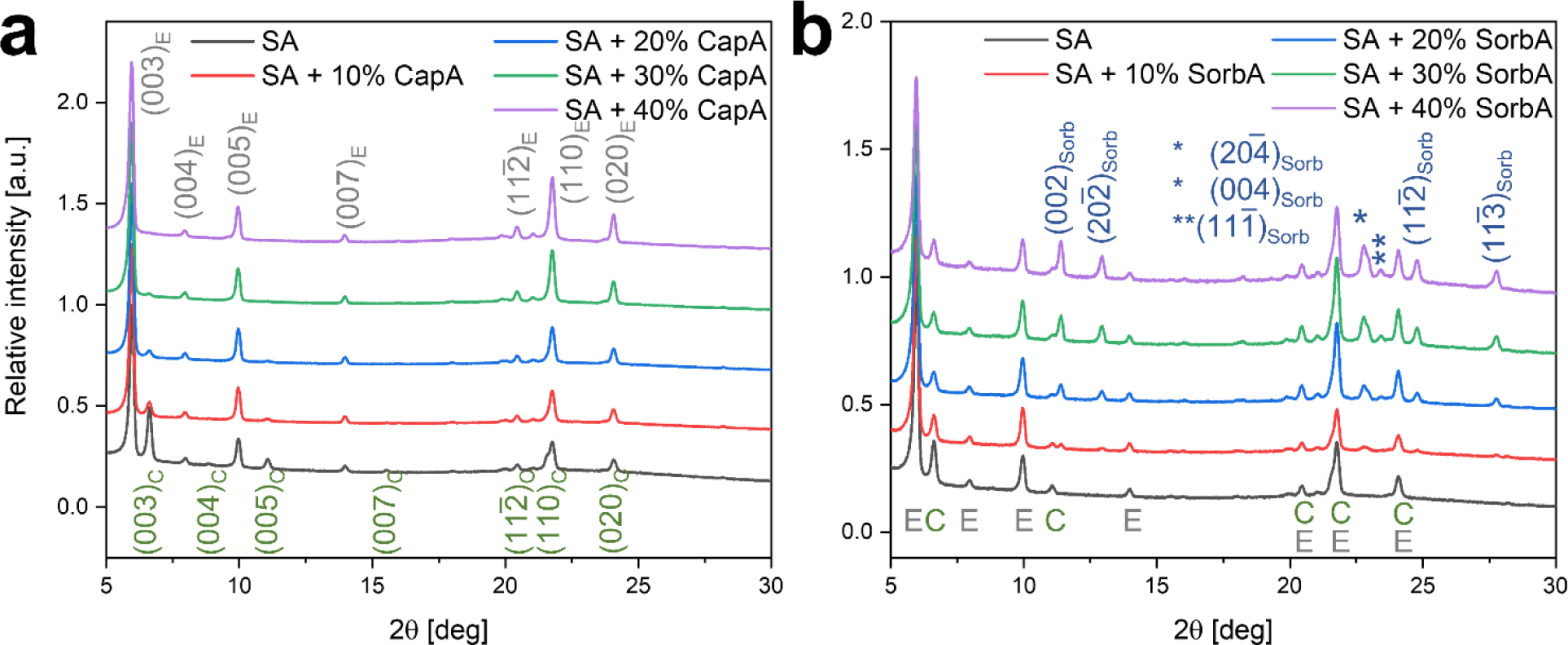
XRD patterns of deposited coatings (λ = 1.54 Å). (a) Stearic acid coatings with 0% (control), 10%, 20%, 30%, and 40% caprylic acid. Two polymorphs of stearic acid are identified: C-form (green, indexed “C”) and E-form (gray, indexed “E”). (b) Stearic acid coatings with 0% (control), 10%, 20%, 30%, and 40% sorbic acid. Stearic acid diffraction peaks are marked with “E” and “C” (indexed as in Figure 2a), with additional diffraction peaks attributed to the sorbic acid phase, marked in blue (“Sorb”).

The addition of caprylic acid influences crystallization, stabilizing the E-form of stearic acid over that of the C-form. In Figure 2a, diffraction patterns show that the intensity of (00l)_C_ peaks associated with the C-form decreases with increasing caprylic acid content. This is particularly evident from the diminishing (003)_C_ peak and the corresponding rise in the intensity of diffraction peaks related to the E-form, such as (003)_E_. This behavior suggests that caprylic acid molecules adsorb at specific crystallographic sites of the stearic acid nuclei, promoting the formation of the E-form polymorph (Figure 1a–e). The analysis of the X-ray diffractions collected from the coatings composed of stearic acid and varying amounts of sorbic acid (Figure 2b) indicates the presence of three phases: sorbic acid and both the E-form and C-form of stearic acid. The intensity of the sorbic acid diffraction peaks increases with its increased amount in the formulation. Unlike caprylic acid, sorbic acid promotes the formation of both the E- and C-form of stearic acid. This difference could be explained by the varying physical states of the MCFAs at RT. Caprylic acid, being liquid, adsorbs onto various facets of the formed stearic acid crystals, affecting the crystallization process and causing a change in the growth rate of either E-form or C-form nuclei types. Sorbic acid, solid at RT, undergoes crystallization alongside the stearic acid crystals; therefore, its effect on stearic acid crystallization is minimal. The XRD analysis of the coatings after 2 months of storage reveals minor structural changes, including the appearance of a peak at approximately 5.6°. This peak does not align with the known polymorphs of stearic acid according to existing literature but may indicate the onset of transformation and crystal reorganization. This new peak is most pronounced in pure stearic acid coatings, while the addition of caprylic and sorbic acids reduces its formation (Figure S2k,l).

High-resolution powder X-ray diffraction (HR-PXRD) using synchrotron radiation (λ = 0.354 Å) was employed to investigate whether the added MCFA molecules are incorporated into the stearic acid crystal lattice. Incorporation of these molecules alters the d-spaces within the lattice, resulting in peak shifts according to Bragg’s law.^63^ After a standard spraying procedure was applied to coat glass substrates with various formulations, the coatings were mechanically detached from the substrate and analyzed in powdered form. The X-ray diffraction patterns of these powdered coatings are shown in Figure S5.

The high-resolution diffraction patterns of the powdered coatings composed of stearic and caprylic acid mixtures corroborate the XRD data collected from the corresponding intact coatings presented in Figure 2, namely, the presence of the two polymorphs of stearic acid, where the addition of caprylic acid favors the formation of the E-form over the C-form (Figure S5a,d). The main reflections of stearic acid that were found in the intact coatings are the (00l)_E_, while (003)_E_ is the strongest reflection; therefore, it was chosen as a representative peak of this direction, and the (110)_E_ reflection, which is related to a different crystallographic direction. The further analysis was focused on these two reflections of stearic acid, as interlattice incorporation (of MCFA) leads to shifts of the reflections related to the matrix (stearic acid). A detailed analysis of the high-resolution diffractograms revealed that in the presence of both caprylic or sorbic acid, no shift in the position of the (003)_E_ diffraction peak is observed (Figure S5b,e). In the case of caprylic acid addition, a slight shift of the (110)_E_ peak to a lower d-spacing is observed, indicating that a minimal inclusion of caprylic acid molecules into the lattice along the direction of stearic acid molecules may take place (Figure S5c). Since the diffraction peak shift is only 0.0035°, corresponding to a 0.07% change in d-spacings, and this shift does not increase as the caprylic acid content rises from 20% to 30%, it can be concluded that the majority of the caprylic acid does not get incorporated within the stearic acid crystals. We would like to emphasize that such small shifts in the diffraction peaks have been demonstrated in other hybrid systems to result from intracrystalline incorporation, such as the inclusion of organic molecules within crystalline calcium carbonate.^63–66^ An analysis of the (003)_E_ peak reveals that the domain size (the average distance between defects) increased by 20–40% with the addition of caprylic acid, indicating a significant increase in the average stearic acid crystal size. This increase may further explain the observed rise in roughness for the caprylic acid-containing samples (details in the discussion following Figure S5). In the case of the sorbic acid addition, a similar shift in the (110)_E_ peak is observed only for the sample with 10% sorbic acid, but not at higher concentrations (Figure S5f). This can be explained by the fact that at low concentrations, sorbic acid is incorporated into the crystalline lattice of stearic acid, whereas at higher concentrations, when the solubility limit is likely exceeded, it segregates as separate crystals.

Fatty acid coatings, being inherently soft, do not possess superior mechanical durability, and particularly, their wear resistance is relatively poor. However, as we demonstrated in a previous study, the fatty acid sprayed coatings undergo thinning upon a “tape test” based on ASTM D3359-17, with only a minor reduction in the WCA; however, the layer does not peel off from the substrate.^31^ The coatings were also found to be stable over time, at elevated temperatures, and when immersed in saline under static and dynamic conditions.^31^ Despite their relatively poor suitability for applications where a high resistance to abrasion is needed, these coatings can be easily used in a wide range of applications where strong mechanical resistance is not required, such as protecting inner surfaces of air conditioning or ventilation ducts and room walls in cellars or cooled storage rooms.

### Application of the Coatings onto Cellulose Filter Paper

In the next step, the feasibility of coating flexible and porous substrates was investigated using cellulose filter paper (Whatman, grade 1, 25 mm, China) as a model surface. Cellulose-based materials, such as paper and wood, are highly susceptible to fungal contamination due to their hygroscopic nature.^67^ Given that superhydrophobic surfaces are well-known for their antimicrobial properties, coating compositions with the highest contact angles and the lowest potential release of MCFAs were selected for this experiment. Specifically, the pure stearic acid coating, along with stearic acid coatings containing 10% caprylic acid or 10% sorbic acid, were chosen. These compositions were sprayed on the paper, and the resulting coatings were characterized for their structure and wetting properties.

Figure 3c presents the confocal micrographs of the coated surfaces, revealing an increased roughness in comparison with the uncoated paper. While the neat uncoated paper, with its characteristic fibrous structure, exhibits a roughness (Sq) of ∼10 μm (Figures 3a,c1 and S6a1), the coated papers display a higher roughness of ∼15–17 μm. This is observed for all three studied compositions and is ascribed to the contribution of the fatty acid crystals to the original roughness derived from the uncoated fibers (Figures 3a, c2,c4 and S6a2–a4).

**Figure 3:**
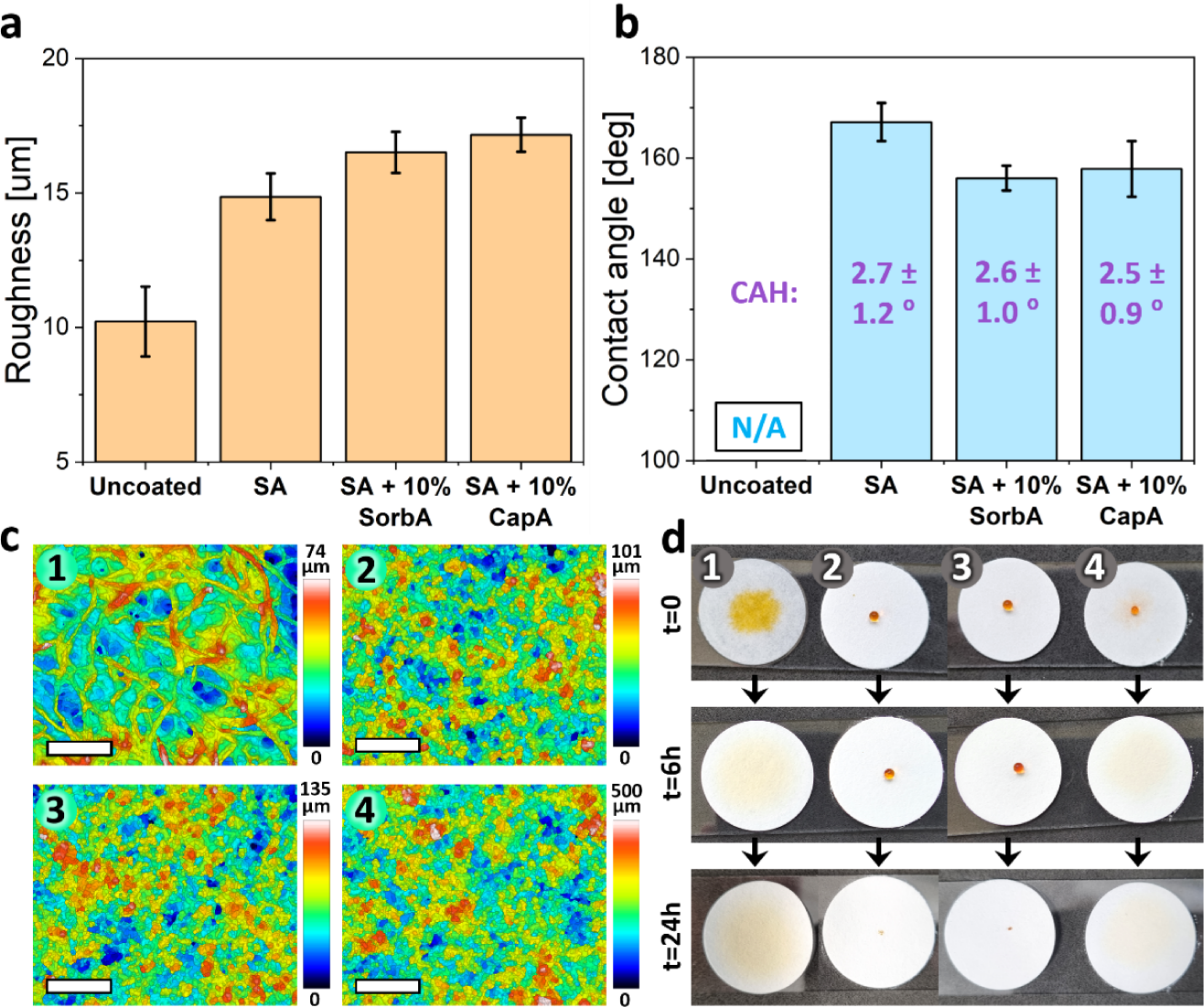
Characterization of fatty acid-based coatings deposited on filter paper. (a) Roughness values. (b) Water contact angles. Values in purple display corresponding contact angle hysteresis. (c) Confocal microscope images of the uncoated and coated paper surfaces: (c1) uncoated paper, (c2) stearic acid coating, (c3) stearic with 10% sorbic acid coating, and (c4) stearic with 10% caprylic acid coating. Scalebar is 200 μm. (d) Digital images of methyl orange-stained water droplets on wet filter papers with different coatings over time: (1) uncoated paper (control), (2) stearic acid coating, (3) stearic +10% sorbic acid coating, and (4) stearic +10% caprylic acid coating.

In all three cases, the coating imparts superhydrophobic properties with self-cleaning ability to the paper surface (CA >156° and CAH <3°) (Figure 3b). To demonstrate the long-term superhydrophobic behavior of the coatings on a water-absorbing substrate, a 7 μL water droplet colored with methyl orange (for easier observation) was placed above the paper substrates. Digital images of the samples, shown in Figure S6b, indicate well-maintained superhydrophobicity over time in the case of both stearic acid and stearic acid with 10% sorbic acid coatings. Stearic acid with a 10% caprylic acid coating exhibits short-term superhydrophobicity, followed by partial absorbance of the water droplet (Figure S6b2). The same experiment was performed using moist paper to subject the coatings to challenging water exposure (see details in the Experimental section), and the wetting properties of the coatings were monitored over time. As shown in Figure 3d, for both stearic acid and stearic acid with 10% sorbic acid coatings, the stained droplets remained unabsorbed even after 6 h, indicating that these coatings prevent water penetration and remain superhydrophobic. After the water evaporated, the residual orange stain, caused by the low contact area between the droplet and the coating, confirmed the stability of the superhydrophobic behavior (Figure 3d, *t* = 24 h)). However, despite the initial superhydrophobic behavior of the stearic acid with 10% caprylic acid coating (Figure S6b1), partial droplet absorption was observed on the moist substrate, even at *t* = 0 (Figure 3d). The higher solubility of caprylic acid, combined with its oily nature that promotes a more homogeneous distribution among the stearic acid crystals, results in reduced coating durability.

### Leaching of MCFAs from the Coatings

The water contact angle values and morphology of the various coatings, as well as the changes in these characteristics over time under ambient conditions, are presented in Figure 1. The wetting properties of the sorbic acid-containing coatings remained stable over time (Figure 1f), whereas those of the caprylic acid-containing coatings changed progressively (Figure 1n), likely due to the gradual release of caprylic acid. The melting point of caprylic acid is significantly lower than that of sorbic acid (*T*_m (stearic acid)_ = 67–69.6 °C, *T*_m (caprylic acid)_ = 16.3–16.7 °C, *T*_m (sorbic acid)_ = 132.8–134.6 °C).^68,69^ This lower melting point leads to much faster diffusion of caprylic acid, which is trapped between stearic acid crystals, to the surface, where it can be released into the atmosphere. Consequently, we investigated the leaching behavior of MCFAs from the coatings.

To this end, the samples were immersed in water under ambient conditions for varying durations to accelerate the MCFAs leaching from the coatings. We note that, due to the significantly higher solubility of both caprylic and sorbic acids in water compared to stearic acid, their release into the aqueous medium is considerably greater over the duration of the experiment (stearic acid: 2.9 × 10^–4^[%], caprylic acid: 0.068[%], and sorbic acid: 0.16[%]).^70,71^ Following immersion, the coated samples were retrieved for characterization, and the composition of the residual aqueous medium was analyzed spectrophotometrically. To facilitate the analysis and prevent rapid release caused by excessive MCFA, 20% MCFA samples were selected from the tested concentration range of 10–40% as the minimum concentration that induced significant changes in both morphology and wetting properties.

Figure 4 summarizes the characterization results for coatings composed of stearic acid with the addition of 20% caprylic or 20% sorbic acids after immersion in water for different time intervals. The surface morphology evolution of the stearic acid coating with 20% caprylic acid is shown in Figure 4a–c. With increasing immersion time, more plate-like crystals emerge from the smooth fatty acid aggregates, leading to the development of a more pronounced hierarchical morphology, similar to that observed in pure stearic acid coatings. Correspondingly, the water contact angle increases from 106° to 163° within the first 15 min of immersion and remains stable upon longer immersion times, up to 24 h (Figure 4d). To confirm the release of caprylic acid, the coating was retrieved, and the composition of the residual aqueous medium was analyzed by absorbance measurements. The characteristic absorption peak of caprylic acid at 265 nm is detected for the caprylic acid-containing samples, with its intensity increasing as the immersion time progresses (Figure 4e). These findings support the hypothesis that shorter, more water-soluble fatty acids leach out of the stearic acid matrix, facilitating the observed increase in the contact angle. The continued rise in the absorption peak intensity of caprylic acid over time further indicates ongoing release, even after the superhydrophobic contact angle stabilizes (after 15 min of immersion).

**Figure 4:**
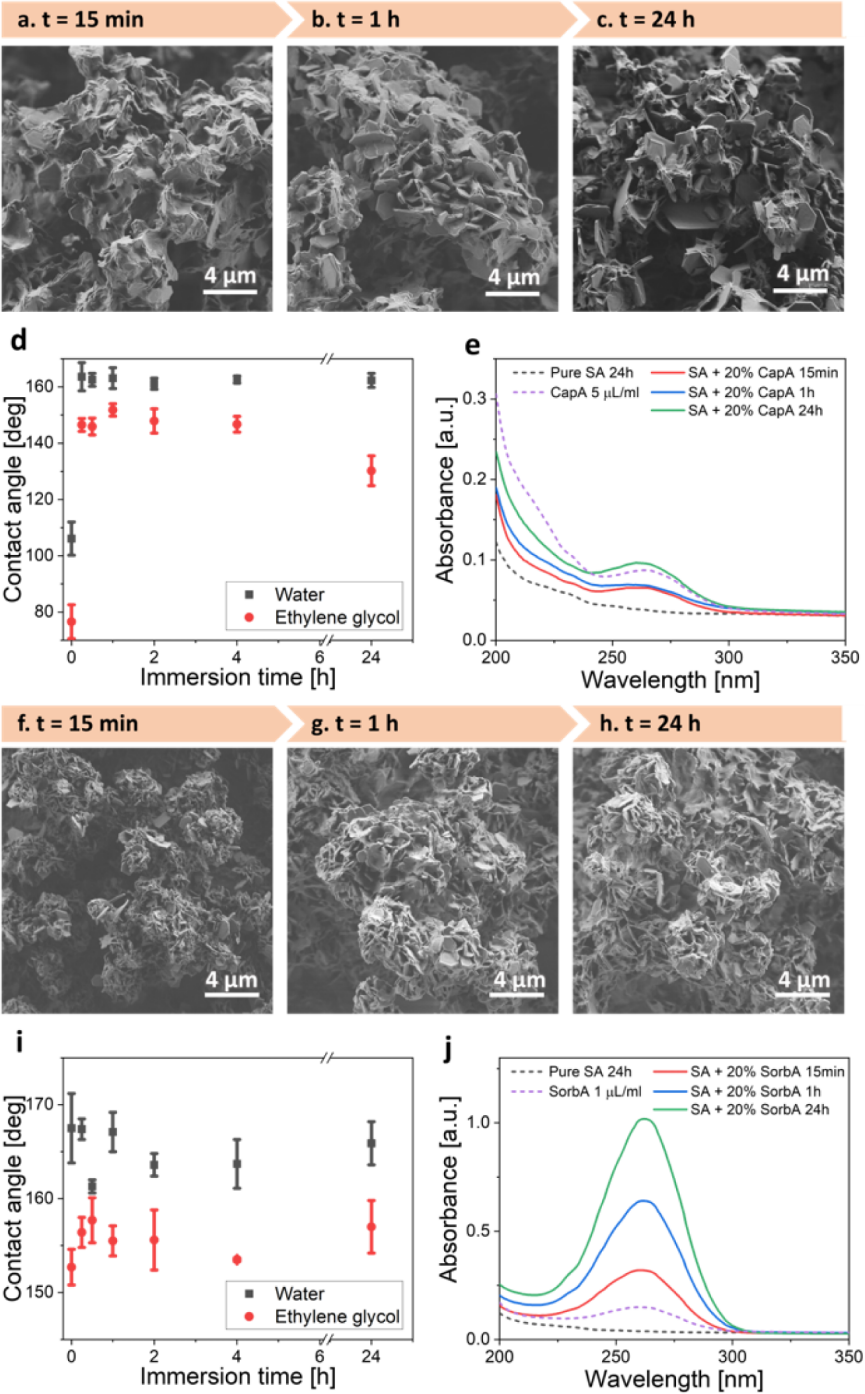
(a–c) HR-SEM images of the coating composed of stearic acid and 20% caprylic acid following immersion in water for: (a) 15 min, (b) 1 h, and (c) 24 h. (d) Changes in water (black) and ethylene glycol (red) contact angle values vs immersion duration. (e) Corresponding absorbance spectra of the collected residual aqueous media following incubation. (f–h) HR-SEM images of the coating composed of stearic acid and 20% sorbic acid following immersion in water: (f) 15 min, (g) 1 h, and (h) 24 h. (i) Changes in water (black) and ethylene glycol (red) contact angle values vs immersion duration. (j) Corresponding absorbance spectra of the collected aqueous media following immersion.

In the case of the stearic acid coating with 20% sorbic acid, the change in crystal morphology upon immersion in water is minimal, consistent with the minor influence of sorbic acid on the stearic acid coating under ambient conditions (Figure 4f–h). The water and ethylene glycol contact angles measured on the postimmersion coatings remain constant within statistical error, even after 24 h. However, similar to the stearic and caprylic acid system, the characteristic absorbance peak of sorbic acid at λ = 265 nm is detected in the water after just 15 min of immersion. The peak intensity increases with immersion time, indicating a gradual release of sorbic acid into the surrounding aqueous medium. This release is further supported by XRD data, which show the disappearance of diffraction peaks associated with sorbic acid after immersion (Figure S7b).

However, the surrounding medium influences the kinetics of surface-related processes, and the aqueous environment likely facilitates leaching of MCFAs from the coating. To conclusively demonstrate the release of caprylic acid under ambient conditions, two additional experiments were conducted. First, TGA analysis was performed in an air environment on the coatings under slightly accelerated conditions at a temperature of 45 °C. Second, the aqueous medium from the immersion experiments, corresponding to samples stored at ambient conditions for various durations, was analyzed using absorbance spectroscopy. Both experiments conclusively support the release of caprylic acid from the coating, even in the absence of an aqueous medium (see details in Figure S8).

### Antifungal Studies

The antifungal activity of the developed single- and multicomponent fatty acid coatings was studied against , known as gray mold. In these studies, mycelial plugs were positioned onto the coated cellulose filter papers (Figure 6a), and mycelial growth was quantified after 72 h.

All coated samples exhibited inhibited mycelial growth in comparison to the uncoated paper (Figure 5a1–a4). We first examined mycelial growth on top of the paper, and for the stearic acid-coated sample, a decrease of >60% in mycelial growth area is detected in comparison to the uncoated sample (Figure 5b, orange bars). Upon the addition of MCFAs, this behavior is further intensified, and coatings containing 10% caprylic acid were found to completely inhibit growth. Characterization of the coatings by optical microscopy (Figure 5c1–c4) further supports these visual observations and also indicates a decrease in the mycelial density developed on the surface of all coatings, where no mycelium was observed for the caprylic acid-containing coating.

**Figure 5:**
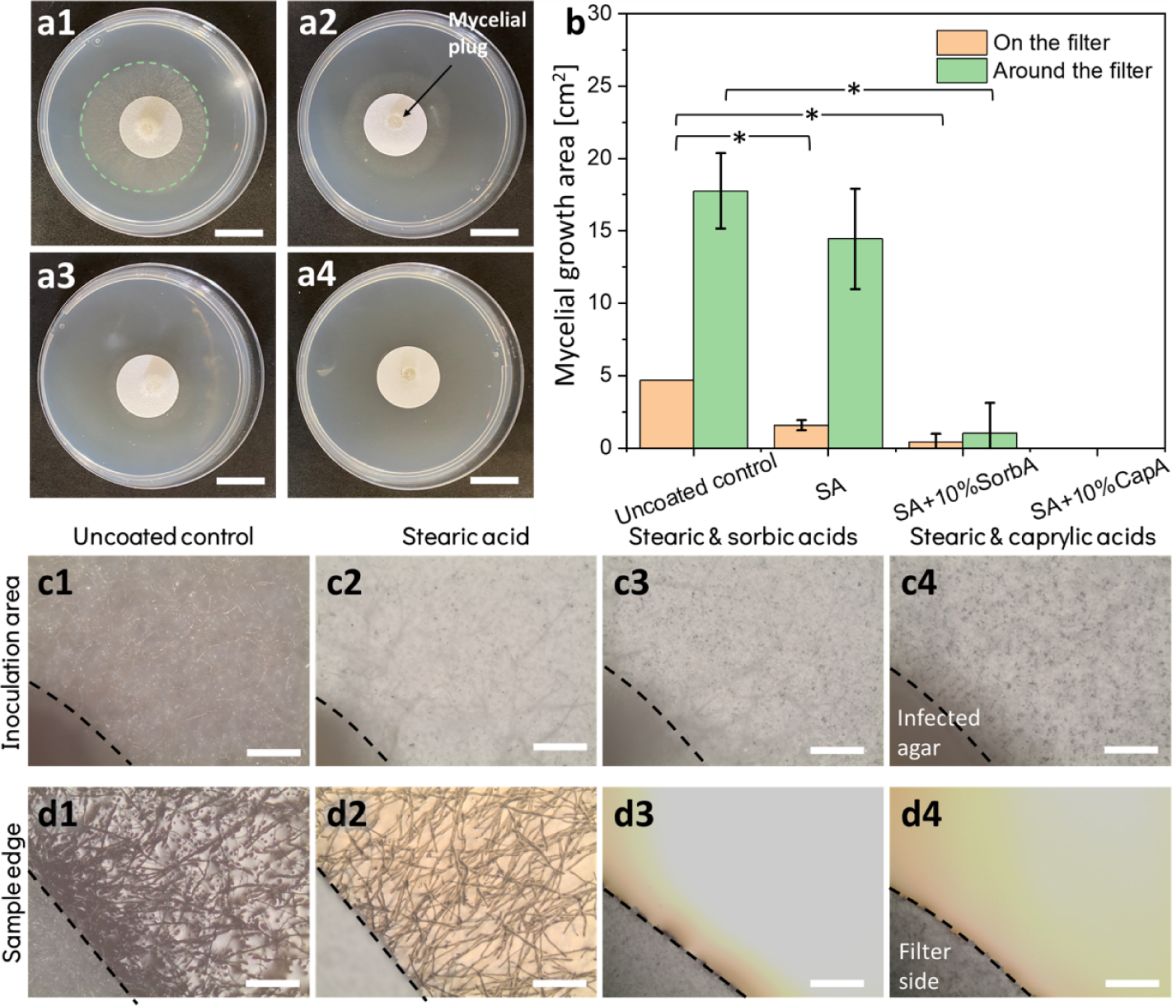
Antifungal activity of fatty acid superhydrophobic coatings. (a) Digital images showing the mycelial growth of on coated papers: (a1) uncoated paper (control, the mycelial growth on the agar is marked for clarity), (a2) stearic acid coating, (a3) stearic with 10% sorbic acid coating, and (a4) stearic with 10% caprylic acid coating. Scalebar: 2 cm. (b) Measured mycelial growth area values for coated and uncoated paper. * indicates significant difference (*p*-value ≤0.05) according to a two-tail test. (c1–c4) Optical microscope images of the mycelial plug area on uncoated control and coated samples (the mycelial plug edges are marked for clarity). Scalebar: 500 μm. (d1–d4) Optical microscope images of the paper edge at the interface with the PDA agar for coated and uncoated samples (the paper edge is marked for clarity). Scalebar: 500 μm.

A similar inhibitory behavior is also observed when inspecting the PDA agar surrounding the paper. The addition of 10% MCFAs is found to significantly inhibit mycelial growth for sorbic acid and completely eradicate the fungi in the case of caprylic acid (Figure 5b, green bars; d1, d3–d4). This intensified growth inhibition effect on the top and around the samples containing MCFAs is expected and attributed to their intrinsic antifungal properties (see details in Figure S9) as well as their diffusion into the surrounding agar upon their release from the coating. However, powdered stearic acid did not lead to any growth inhibition (Figure S9b), and yet, in the form of a superhydrophobic coating, it significantly reduced growth. The growth inhibition is well observed on top of the coated paper (Figure 5b, orange bars; parts c1–c2). Moreover, the density of the mycelium network and the number of infection cushions^72^ are reduced when comparing the morphology of the mycelium surrounding the stearic acid coating to that developed around the uncoated paper (Figure 5d1–d2).

Electron microscopy was used to further investigate the morphological changes in the mycelium structures developed on the coatings. Figure 6b1 reveals that the mycelium growing on top of the stearic acid-coated sample presents fewer hyphae in comparison to the dense, well-branched network that developed on the uncoated paper (Figure 6a1). The hyphae are observed to extend between the stearic acid crystals and display a lower degree of invasive hyphal apical branching, limiting their ability to spread and colonize.^73,74^ This behavior is also observed in the case of the stearic acid coating containing 10% sorbic acid, where sporadic entangled hyphae are detected (Figure 6c1). No growth of was observed in the case of coatings containing 10% caprylic acid, in agreement with the optical microscopy results.

**Figure 6:**
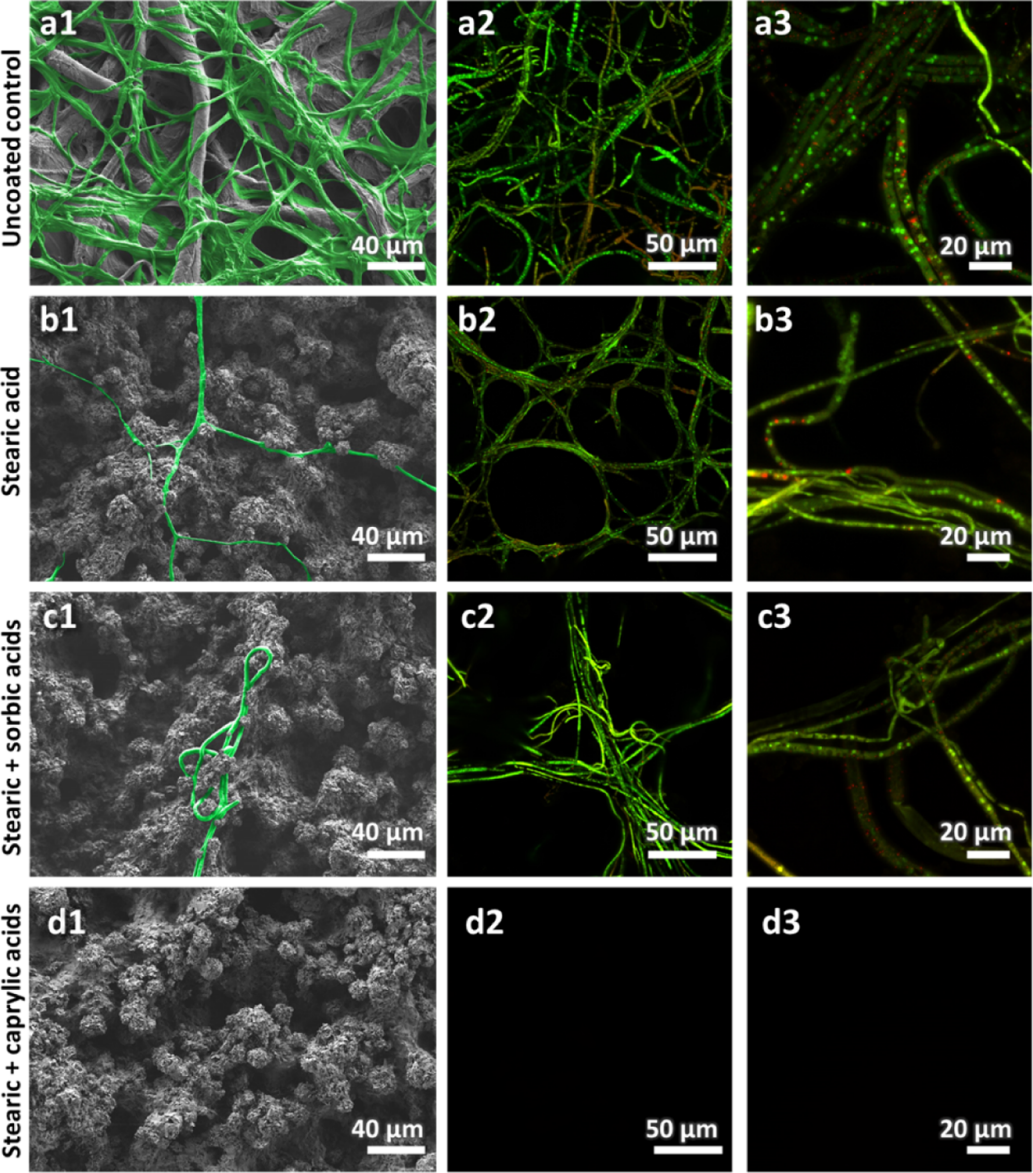
Microscopy images of that developed on the paper substrate after 72 h of incubation at 24 °C. (a1–d1) HR-SEM micrographs depict grown on uncoated paper, stearic acid-coated paper, stearic with 10% sorbic acid-coated paper, and stearic with 10% caprylic acid-coated paper, respectively. The mycelium is false-colored green for clarity. (a2–d2) LSCM images show stained with FUN-1 dye on the same corresponding papers. (a3–d3) Zoomed-in LSCM images present a closer look at after Fun-1 staining on the different coatings.

To investigate the effect of the coatings on fungal viability, the samples were stained with FUN-1, a membrane-permeable fluorescent dye^60^, and subsequently imaged. While the dye is internalized by both living and dead cells with intact plasma membranes, resulting in a diffuse green fluorescence within the cytoplasm, only in metabolically active cells is the green fluorescence converted into red cylindrical intracellular structures (CIVS), providing an indicator of cell viability.^57^ Figure 6a2,a3 depicts CLSM images of the uncoated paper, where the fungus is observed to produce abundant and well-formed hyphae in which numerous red CIVS are detected, confirming fungal viability. In the case of both stearic acid and stearic acid with 10% sorbic acid coatings (Figure 6b2,b3,c2,c3), fewer CIVS are observed, indicating a significant reduction in metabolic activity and fungal viability.^75^ For the coating containing 10% caprylic acid, no fluorescence is detected (Figure 6d2,d3) which is consistent with both optical and electron microscopy studies.

These results demonstrate that the antifungal activity of the coatings can be tuned from passive inhibition of fungal adhesion and colonization onto the surface in the case of pure stearic acid coatings to biocidal activity in the case of MCFAs addition to the coatings, where complete eradication of the fungus can be realized. Superhydrophobic surfaces are well-known for their antibiofouling properties, particularly against bacteria.^14,76^ In most cases, the surface’s roughness minimizes the contact area available for pathogen cells, thereby reducing their adhesion and colonization. In our previous study, we have demonstrated that superhydrophobic coatings based on single fatty acids, such as stearic acid, exhibit a unique combination of antibiofouling and antibacterial properties against common bacteria.^31^ The coatings’ selective activity against different bacteria is ascribed to their complex hierarchical structure and the fatty acid’s intrinsic biocidal effect.^33^ In this study, the MCFAs act as active biocidal agents (Figure S9c,d) that are incorporated into superhydrophobic stearic acid coating, which expresses its own passive antifungal behavior. These results agree with previous studies in which the antimicrobial properties of the studied MCFAs against numerous fungal species were demonstrated, as well as toward .^46,77^ The antifungal properties of these fatty acids are primarily attributed to their ability to partition within the lipid bilayer of the fungal membranes, increase their fluidity, and hence disrupt their integrity and function.^46^ In addition, their antimicrobial action is also exerted by targeting various cellular functions, such as inhibition of topoisomerase, accumulation of reactive oxygen species, and interference with fatty acid biosynthesis.^46^ However, the limited amount of MCFAs in the coating and their gradual leaching are expected to provide only a short-term antifungal effect, whereas the pure stearic acid superhydrophobic coating acts passively, with its effectiveness not relying on the incorporation of potent MCFAs. This is evidenced by the reduced antifungal activity of aged coatings containing MCFAs. As shown in Figure S10, 8-day-old stearic acid coatings with 10% caprylic acid allowed visible mycelial growth, in contrast to the complete inhibition observed with freshly prepared coatings. Similarly, increased mycelial growth was observed for aged sorbic acid-containing coatings. Importantly, the aged stearic acid coating inhibits the mycelial growth in comparison to the noncoated samples. Therefore, in scenarios where a nonleaching, passive mode of antifungal action is preferred, a single-component stearic acid coating may be advantageous.

We propose that the superhydrophobicity and the multiscale topography of the stearic acid-based coatings are not only unfavorable for the adhesion of fungal spores but also disrupt the formation of stable mycelium.^78–80^ This disruption leads to significant growth inhibition and notable alterations in mycelium morphology, particularly a reduced degree of branching. This reduction in branching adversely affects nutrient uptake and oxygen consumption, further impeding fungal growth.^74^ Given that proper hyphal branching is crucial for the pathogenicity of , the observed decrease in branching may also account for diminished virulence and reduced colonization capacity.^73^ When 10% caprylic or sorbic acids are incorporated within the stearic acid coating, the hierarchical morphology and superhydrophobicity of the coatings are generally preserved. These potent MCFAs gradually leach out into the surrounding environment, exerting their antifungal effects through the mechanisms discussed earlier.^47^ This, in turn, results in reduced fungal viability in the case of sorbic acid and complete inhibition for caprylic acid. Indeed, numerous studies have reported the superior antifungal activity, including fungicidal effects, of free caprylic acid against various types of filamentous fungi.^80,81^ To summarize, the proposed superhydrophobic single-component stearic acid coating can be used for passive prevention of fungal growth, while the initial antifungal effect can be intensified with the addition of potent MCFAs.

## Conclusions

In this study, we developed multifunctional superhydrophobic coatings composed of fatty acids with the capability of releasing active ingredients. These stearic acid coatings demonstrate excellent superhydrophobicity and self-cleaning abilities, even with the incorporation of water-soluble sorbic acid. They can be applied to substrates with different physical properties, including flat glass and water-absorbing cellulose rough paper with complex fiberlike morphology, and remain durable even on fully wet substrates. We investigated the incorporation of MCFAs with varying chemical and physical properties into the coating and examined how the properties of the coating are affected when using solid or liquid fatty acids. Sorbic acid (solid) can be added in higher amounts with minimal impact on the coating’s properties, whereas caprylic acid (liquid) significantly reduces superhydrophobicity and alters the morphology of the coatings. We demonstrated that the incorporated MCFAs can diffuse out of the stearic acid matrix, making them available as free compounds in the surrounding environment. The leaching rate is environment-dependent, with accelerated release in aqueous conditions. Notably, as caprylic acid is released from the matrix, its influence on the coating properties diminishes, allowing the recovery of the coating’s superhydrophobic characteristics.

The antifungal activity of the sprayed fatty acid coatings against was demonstrated for all studied formulations, including the pure stearic acid coating (without MCFAs). The incorporation of MCFAs further enhances the antifungal performance. The resulting coatings exhibit bimodal functionality: a passive inhibitory effect provided by the superhydrophobic stearic acid coating, combined with the potent fungicidal action of the MCFAs embedded within the matrix. Thus, we suggest that while the leaching of caprylic acid provides an initial pronounced antifungal effect, the resulting increase in the coating’s superhydrophobicity ensures sustained antifungal performance.

## Supplementary Material



## Notes

The supplementary data include the composition of the spray solutions, roughness parameters for the various coatings, and detailed two-month stability data (featuring HR-SEM images and XRD analysis). Additional characterization encompasses wide field-of-view HR-SEM images of stearic acid and stearic/sorbic acid coatings, XRD data for powdered stearic acid, and HP-XRD measurements with theoretical discussion for the powdered coatings. The coatings applied on cellulose filter paper are characterized using both HR-SEM and digital imaging. The data set also covers XRD analysis after water immersion, evaluation of caprylic acid release at ambient conditions (using TGA and absorbance measurements), investigation of the intrinsic antifungal properties of the studied fatty acids (digital images), and assessment of the antifungal activity of 8-day-old fatty acid coatings (PDF)
