# Supplementary material for: Superhydrophobic Fatty Acid-Based Spray Coatings with Dual-Mode Antifungal Activity
Source: ACS Appl Bio Mater. 2025 Jun 10;8(7):5970–83. doi: 10.1021/acsabm.5c00596 (PMC12284891; doi:10.1021/acsabm.5c00596)
Supplement: Supplementary file 1 [file mt5c00596_si_001.pdf]

Supporting Information

Superhydrophobic Fatty Acid-Based Spray Coatings with Dual-mode Antifungal Activity

Elena Prudnikov [a], Hanan Abu Hamad [b], Iryna Polishchuk [a], Alexander Katsman [a],

Ester Segal [b] and Boaz Pokroy [a*]*

[a*] Department of Materials Science and Engineering, Technion − Israel Institute of Technology, 3200003 Haifa, Israel. E-mail: bpokroy@technion.ac.il; Tel: +972-4-829-4584

[b] Faculty of Biotechnology and Food Engineering, Technion − Israel Institute of Technology, 3200003 Haifa, Israel

KEYWORDS: superhydrophobic, self-cleaning, coatings, fatty acids, spray coating, active components incorporation and release, antimicrobial, antifungal

Composition of the solutions

The composition of solutions used to form various coatings is detailed in Table S1. The caprylic acid volume was calculated by division of the required weight (the same as for sorbic acid) by the density – 0.910 g/mL.

Table S1: Composition of spray solutions used to form multi-component coatings

MCFA concentration in the coating [%]	Stearic acid weight in 50 mL of 0.02 g/ mL solution [g]	Sorbic acid weight (±5 mg) [mg]	Caprylic acid volume [µL]
10	1	111	122.1
20	1	250	274.7
30	1	429	471.0
40	1	667	732.6

Roughness and surface parameters:

Root mean square values (Sq) shown in Figure 1 provide the information on vertical dimensions of the surface. For better understanding of the fabricated surfaces, roughness parameters addressing the horizontal surface dimensions[1], namely developed area ratio (Sdr, Figure S1 a, d) and density of peaks (Spd, Figure S1 b, e) were tested, as well as the skewness (Ssk), which is related to the histogram of height distribution of the scanned surface (Figure S1 c, f). In both cases, coatings with caprylic or with sorbic acid, the Sdr and Spd values follow the same trend that shown for Sq: for higher roughness, also higher values of the developed area ratio and the peaks density were obtained. The sample with 10% caprylic acid expresses the same roughness value, but slightly higher Sdr and Spd values in comparison to the 20% caprylic acid. The higher values of the developed area ratio and the peaks density may be addressed to less prominent smoothing of the surface features that increase with the addition of

caprylic acid to the formulation. Low positive values of the skewness indicate relatively narrow surface features that formed on the supporting substrate.

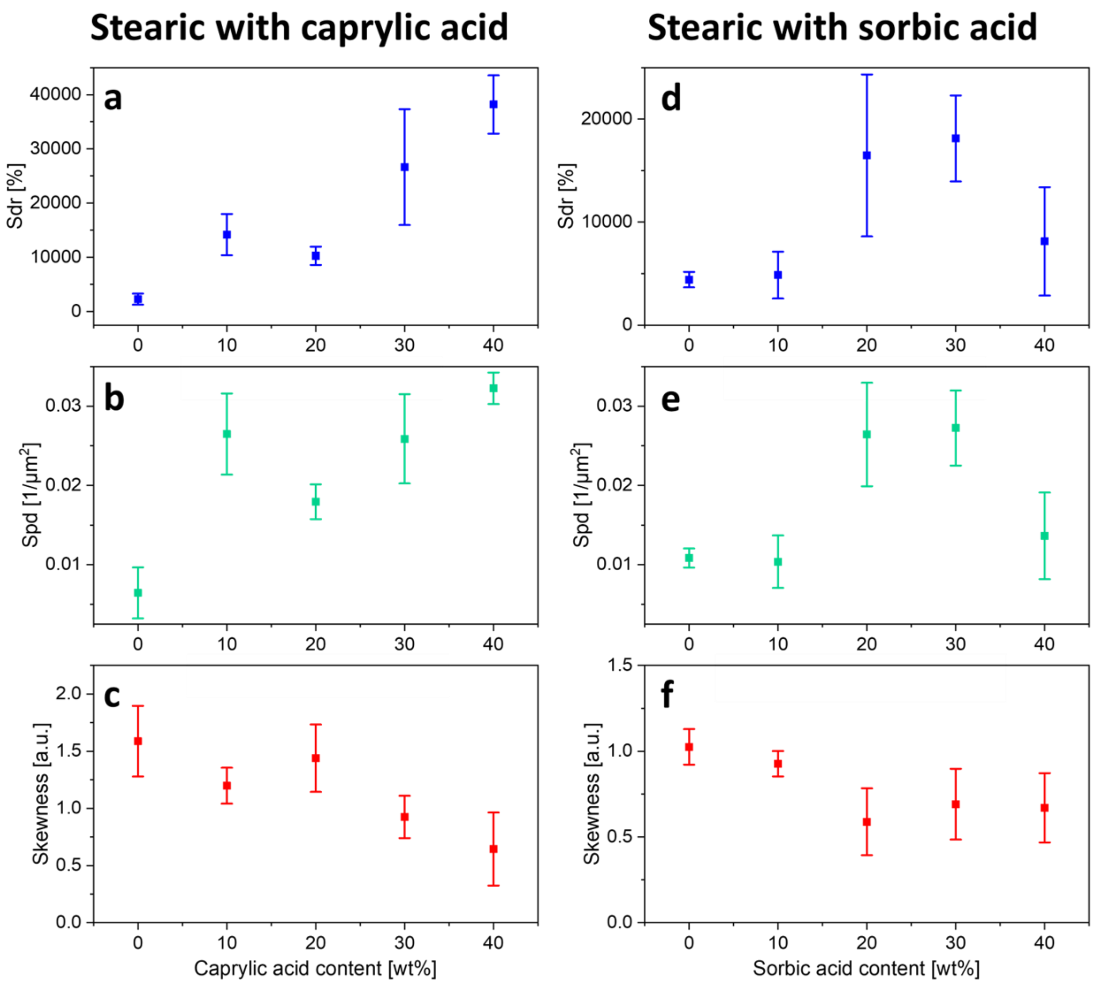

Figure S1: Roughness parameters derived from confocal microscope measurements. (a-c) Caprylic acid containing coatings, (d-f) Sorbic acid containing coatings. (a, d) Developed area ratio (Sdr), (b, e) Density of peaks (Spd), (c, f) Skewness (Ssk).

<u>2-months stability of the coatings:</u>

HR-SEM images and X-ray diffractions of the coatings were obtained after 2 months of storage at ambient conditions. The results are shown in Figure S2:

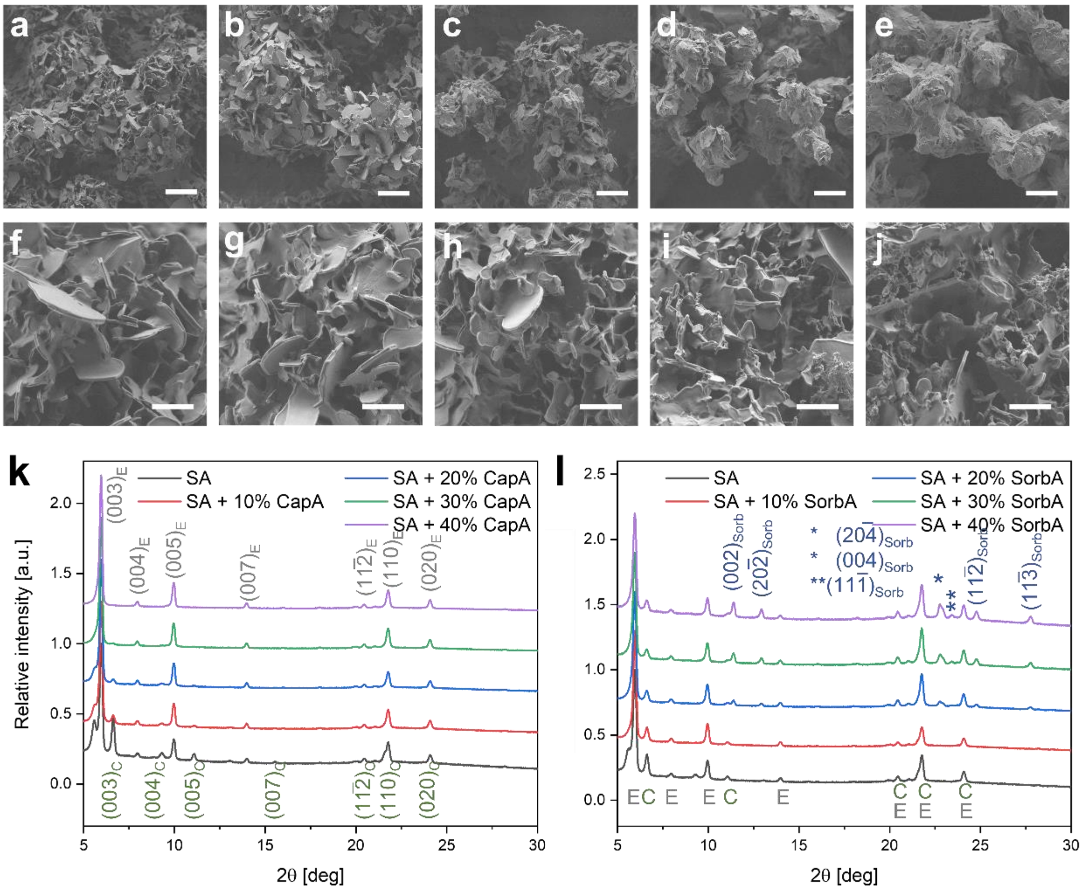

Figure S2: The coatings after 2 months of storage: (a-e) HR-SEM images of sprayed stearic acid coating with increasing amounts of caprylic acid – 0%, 10%, 20%, 30%, and 40% caprylic acid, respectively. Scalebar is 6 µm. (f-j) HR-SEM images of sprayed stearic acid coating with increasing amounts of sorbic acid – 0%, 10%, 20%, 30%, and 40% of sorbic acid, respectively, scalebar is 2 µm. (k) XRD of the stearic acid coatings with the addition of 0% (control), 10%, 20%, 30% and 40% of caprylic acid. (l) XRD of the stearic acid coatings with the addition of 0% (control), 10%, 20%, 30% and 40% of sorbic acid.

Contrary to a significant change in the wetting properties of stearic with caprylic acid coatings over time (Figure 1 f-g), no significant morphological changes were found after two months of storage at ambient conditions (Figure 1 a-e vs. Figure S2 a-e). The possible evaporation of caprylic acid from the surface improves the hydrophobicity due to the chemical change of the outer coating layer and causes an increase in the contact angle. Still, the formed crystals cannot be easily reorganized and the characteristic morphology of the pure sprayed stearic acid is not recovered. The morphology of the coatings formed from out of stearic with sorbic acid remains stable over the tested period (Figure 1 i-m vs. Figure S2 f-j). The XRD of the coatings after 2 months of storage at ambient conditions for both coating series of stearic acid with caprylic or with sorbic acid does not show significant changes, except for an appearance of peak at ~5.6°. This peak does not accurately fit the polymorphs of stearic acid but may indicate the beginning of transformation and crystal reorganization. This new peak

appearance is most prominent in pure stearic acid coatings, and adding both caprylic and sorbic acids decreases its formation.

Wide FoV of stearic with sorbic acid coatings:

HR-SEM images of stearic with sorbic acid coatings at similar magnification as stearic with caprylic acid coatings shown in Figure 1 a-e:

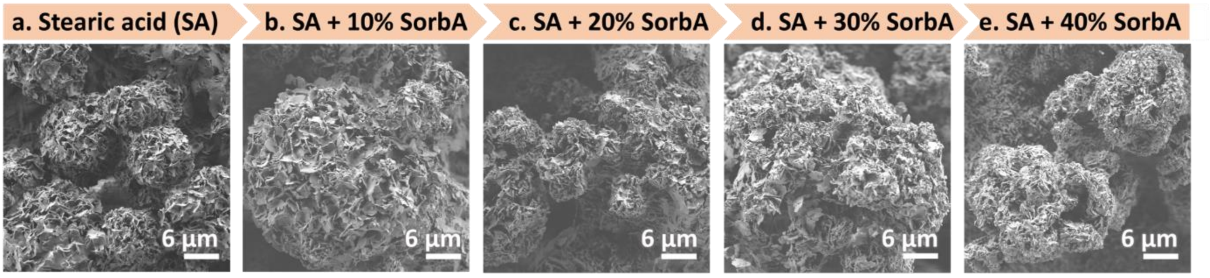

Figure S3: HR-SEM images of sprayed stearic acid coating with various amounts of sorbic acid (SorbA) – 0%, 10%, 20%, 30%, and 40%, respectively

X-ray diffraction of powdered stearic acid:

Figure S4 shows the diffraction patterns of powdered stearic acid and sprayed stearic acid coating. The spray processing results in a preferred orientation of the (00l) plane and a prefferable formation of the E-form polymorph.

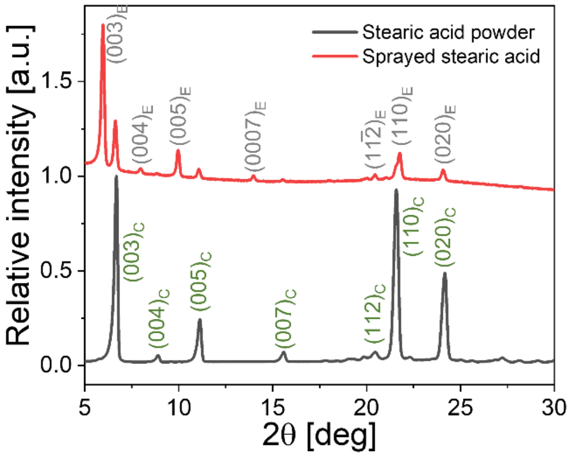

Figure S4: XRD of powdered stearic acid and stearic acid coating formed by spray coating.

HR-PXRD of the powdered coatings:

The high-resolution diffractions of the powdered coatings (HR-PXRD) of stearic with caprylic acid coatings support the obtained data from the XRD of the intact coatings shown in Figure 2.

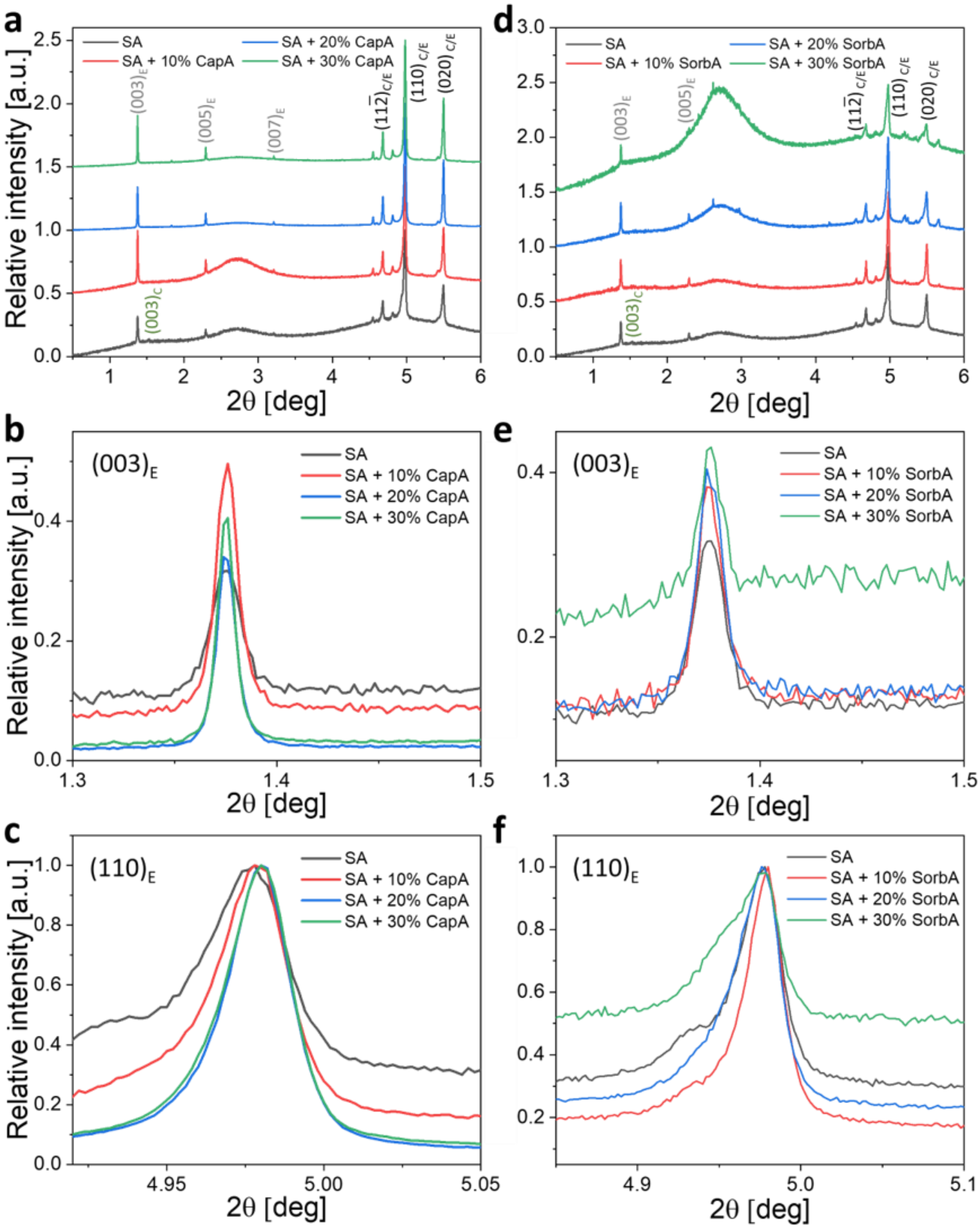

Figure S5: HR-PXRD of detached powdered coatings performed with λ=0.3542 [Å]: (a-c) Detached powdered coatings of stearic with caprylic acid. (a) Diffraction of 0.5-6 [deg] range. (b) Zoom-in on the $(003)_E$ reflection. (c) Zoom-in on the $(110)_E$ reflection. (d-f) Detached powdered coatings of stearic with sorbic acid. (d) Diffraction of 0.5-6 [deg] range. (e) Zoom-in on the $(003)_E$ reflection. (f) Zoom-in on the $(110)_E$ reflection.

The increase in roughness of the caprylic acid containing samples may also be associated with an increase in the average size of stearic acid crystals, which is manifested in an increase in the average domain size (the distance between defects in the crystal lattice). The domain size was calculated according to Scherrer equation, when FWHM of the $(003)_E$ peak of stearic acid was analyzed (Figure S5 b):

$$\tau = \frac{K\lambda}{\beta\cos(\theta)} \tag{1S}$$

where τ is the mean size of the ordered (crystalline) domains, λ is the radiation wavelength (λ=0.3542Å for synchrotron radiation), β is the peak broadening at half the maximum (FWHM), and θ is the Bragg angle.

According to this analysis, the domain size increases in the caprylic acid containing samples by 20%-40%. Corresponding increase in the average size of stearic acid crystals can be explained by a decrease in the nucleation rate (*J*) of stearic acid crystals, when crystallization starts from a solution containing both stearic and caprylic acid:

$$J \sim \exp(-\Delta G / k_B T) \tag{2S}$$

where ΔG is the free energy barrier for nucleation, k_B is the Boltzmann constant, T is the absolute temperature; according to classical theory of nucleation[2]:

$$\Delta G = k\gamma^3 / \Delta G_v^2 \tag{3S}$$

where γ is the nucleus surface tension, ΔG_v is the Gibbs free energy change during solidification of stearic acid per unit volume, *k* is a dimensionless geometry factor. The ΔG_v represents the gain in free energy during the transition of stearic acid molecules from a liquid solution to a nucleated crystal of stearic acid, and equals to the difference between the free energy of the stearic acid molecules in the crystal, ΔG_v^{crys}, and their free energy in the solution, ΔG_v^{sol}:

$$\Delta G_v = \Delta G_v^{crys} - \Delta G_v^{sol} \tag{4S}$$

It should be noted that all these free energies are negative, and ΔG_v^{crys} is more negative than ΔG_v^{sol}, which is the driving force for crystallization. It is quite reasonable to assume that the presence of caprylic acid molecules in the solution leads to decrease of the ΔG_v^{sol} (making it more negative) due

to substantial interaction of stearic and caprylic molecules present in the solvent - diethyl ether; interactions of stearic acid molecules with caprylic acid molecules is stronger than that with molecules of diethyl ether due to stronger hydrogen bonding (two carboxylic acids vs. one carboxylic acid in an ether) and due to additional hydrophobic interactions from the long alkyl chains of stearic and caprylic acid which contribute to van der Waals interactions. Decreasing G_v^{sol} (and therefore increasing in absolute value) results in decrease of ΔG_v, leading to increase of the free energy barrier for nucleation ΔG, eq.(3S), and corresponding decrease of the nucleation rate, eq.(2S). It results finally in fewer stearic acid crystals of larger size. Since the final total number of crystals, N, is proportional to the nucleation rate, J, an average size of crystals can be estimated as:

$$\bar{D} = L / N^{1/3} \sim \exp\left(\frac{\Delta G}{3k_B T}\right) = \exp\left(\frac{k\gamma^3}{3\Delta G_v^2 k_B T}\right) , \qquad (5S)$$

where L is the linear size of the solidified system. So, the average size increases exponentially with decrease of ΔG_v. These larger (and in smaller number) crystals then participate in formation of smoother spheres, which is provided by liquid layers of caprylic acid. In contrast, spheres formed from the pure stearic acid solution are comprised of a high number of separate plate-like crystals.

Characterization of the coatings applied on cellulose filter paper:

The application feasibility and performance of the pure stearic acid coating and multi-component stearic acid coating containing 10% caprylic or 10% sorbic acid were tested when applied on filter paper and their HR-SEM images were obtained (Figure S6 a1-a4). The coatings' morphology is similar to the obtained morphology when deposited on glass slides (Figure 1 a, b, I, j). Higher heterogeneity of crystal distribution can be seen as a result of the fiber-like substrate structure, supporting the obtained results from the dynamic confocal microscope scans (Figure 3 c).

To test the wetting properties of the coating when water-absorbing filter paper acts as a substrate, a methyl orange-colored water droplet of 7 µL was placed above the samples, covered with a petri dish to avoid evaporation, and followed over time. The results are shown in Figure S6 b1-b3:

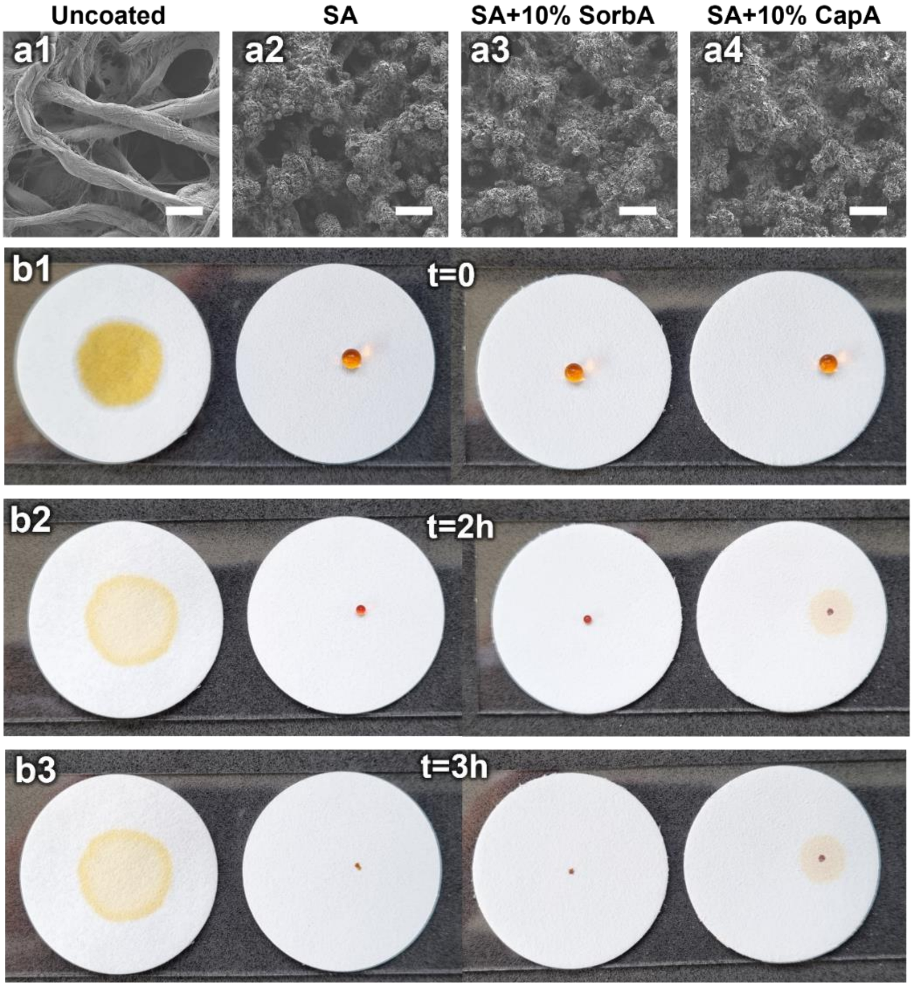

Figure S6: (a1-a4) HR-SEM images of uncoated paper and different coatings deposited on paper. (a1) Uncoated paper. (a2) Stearic acid coating. (a3) Stearic with 10% sorbic acids coating. (a4) Stearic with 10% caprylic acids coating. Scalebar is 30 µm. (b1-b3) Methyl orange-stained water droplet on dry samples over time. (b1) At t=0. (b2) After 2 hours. (b3) After 3 hours. Samples from left to right: uncoated paper, stearic acid coated paper, stearic with 10% sorbic acid coated paper, stearic with 10% caprylic acids coated paper.

The water droplet is not absorbed and remains stable over 2 hours on pure stearic acid coating and stearic with 10% sorbic acids coating (Figure S6 b1, b2). In the case of stearic with 10% caprylic acid coating, partial water absorption into the paper through the coating takes place, but after 2 hours, an unabsorbed droplet still exists (Figure S6 b1, b2). Despite the cover, the droplets completely evaporated after 3 hours, and the remaining methyl orange spots indicate the low contact area between the droplet and the coating. That result proves the stable superhydrophobicity of stearic acid coating and stearic acid with 10% sorbic acid coating over time (Figure S6 b3).

XRD of the coatings after immersion in water:

XRD of different coatings after immersion in water are shown in Figure S7:

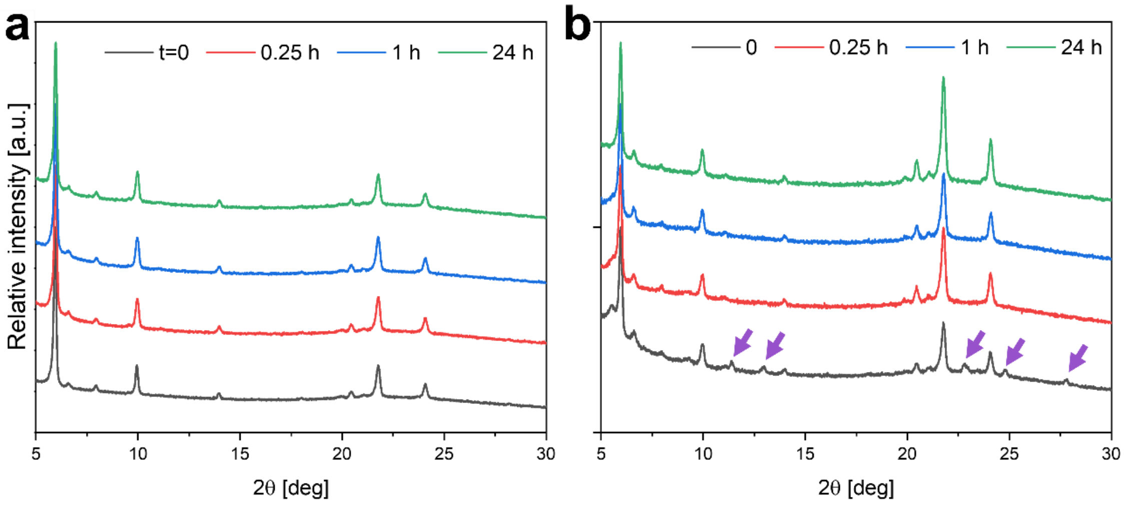

Figure S7: XRD of coatings after immersion in water for 15 min, 1 h and 24 h. (a) Stearic with 20% caprylic acid coating. (b) Stearic with 20% sorbic acid coating.

Despite the evolution of the morphology upon immersion of the coating with caprylic acid in water (Figure 4 a-c) and the evidence of caprylic acid release from the coating (Figure 4 e), no significant change was found in the structure (Figure S7 a). In the case of sorbic acid addition, the peaks related to the sorbic acid disappear after immersion in water for 15 min (Figure S7 b, purple arrows). The increasing absorbance peak of the analyzed water (Figure 4) indicates the continuation of the release of sorbic acid from the coating from deeper zones that are probably not reached by X-ray in the measurement setup.

Evaluation of caprylic acid release at ambient conditions:

To prove our assumption that caprylic acid is released to the atmosphere during storage at ambient conditions, an accelerated conditions study was performed using TGA analysis. Freshly prepared samples of pure stearic acid, or stearic with 20% caprylic acid were mechanically detached from the glass substrate and the weight loss at 45°C with constant air flow was measured. Figure S8 a show the weight loss as a result of caprylic acid release over time, with calculated weight decrease of 10.4% over 10 hours. No weight loss was detected for the pure stearic acid sample (Figure S8 c). Subsequently, high-resolution TGA was implemented for determination of the remaining caprylic acid in the sample after 10 hours at 45°C. The results shown in Figure S8 b indicate a corresponding weight loss of 10.7% related to evaporation of caprylic acid before the main weight loss related to stearic acid evaporation occurs (see Figure S8 d for pure stearic acid sample).

To assure the release of caprylic acid to the atmosphere during storage at ambient conditions in non-accelerated study, identical samples (round, 18 mm in diameter glass slides, coated with stearic + 20% caprylic acid) were immersed in 5 ml of double-distilled water for 1 h after different storage periods: freshly prepared (t=0), after 3 days, and after 11 days of storage. After 1 hour of immersion, 200 µL aliquots of the aqueous medium were collected and absorbance measurements were carried out. The results show a decrease in the absorbance intensity of the peak at 265 nm (characteristic of caprylic acid), for samples that stored for a longer period prior to immersion in water, indicating a lower concentration of caprylic acid after longer storage (Figure S8 e). This experiment suggests that a lower content of caprylic acid remains in the in the coating after storage at ambient conditions.

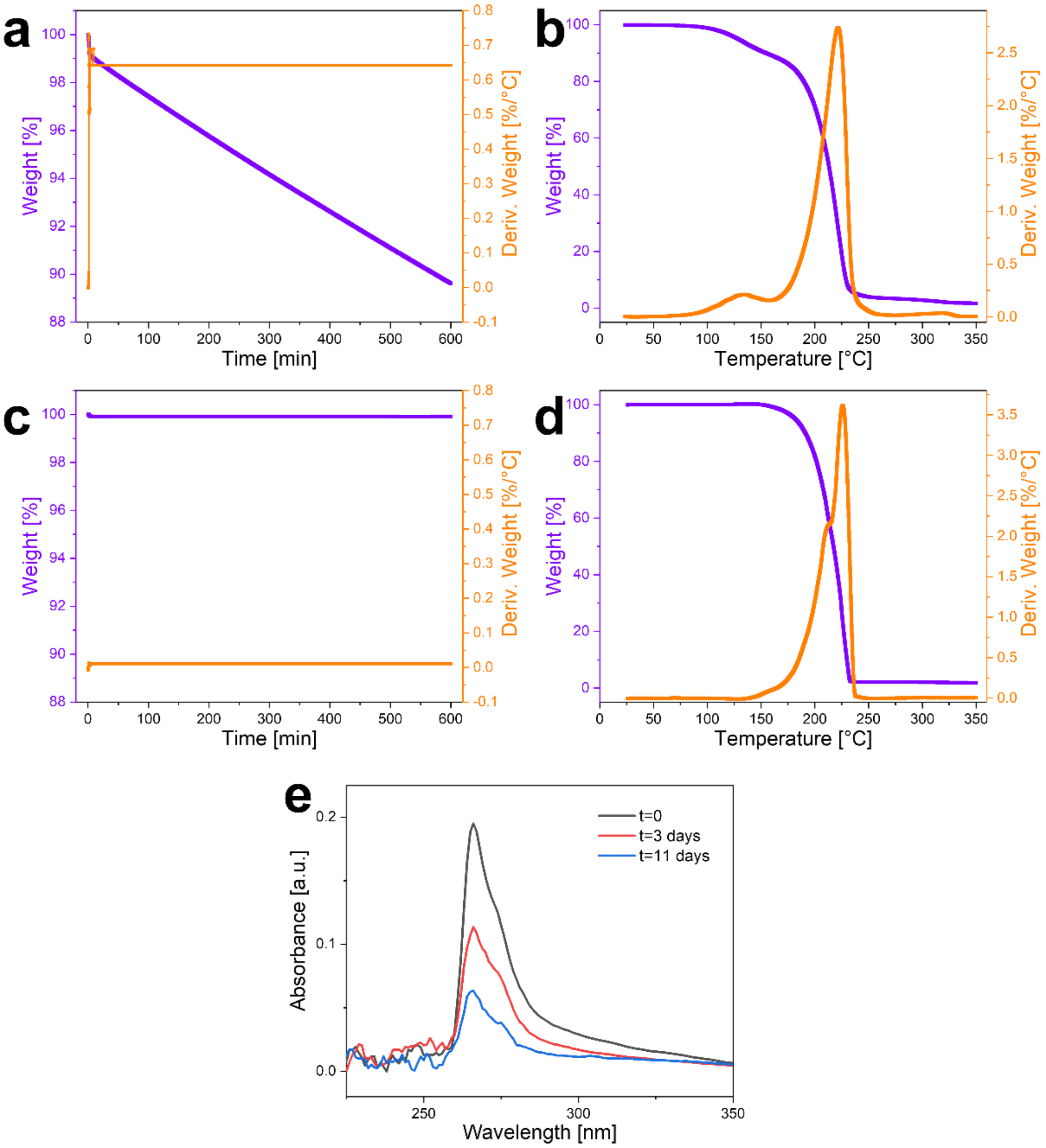

Figure S8: Thermogravimetric analysis of coatings: (a,c) Thermograms and corresponding (first derivative) dTGA curves of detached stearic acid with 20% caprylic acid and stearic acid coatings, respectively, under isothermal conditions of 45°C for 10 hours under air atmosphere. (b,d) Dynamic high-resolution TGA and dTGA results for the stearic acid with 20% caprylic acid and stearic acid coatings, respectively, subsequently performed after the isothermal step (10 hours at 45°C). (e) Corresponding absorbance spectra of the collected aqueous media following 1-hour immersion of the stearic acid with 20% caprylic acid coating, stored for different periods at ambient conditions.

<u>Investigation of intrinsic antifungal properties of the studied fatty acids:</u>

To elucidate the mode of action of our coatings against *B. cinerea*, we have characterized the intrinsic antifungal activity of the selected fatty acids in their native form (as powder or a liquid) by the agar diffusion method. To this end, 50 mg of each fatty acid was placed in the center of a petri dish containing *B. cinerea*-infected agar. The samples were incubated for 7 days at 24°C, and mycelium growth was examined.

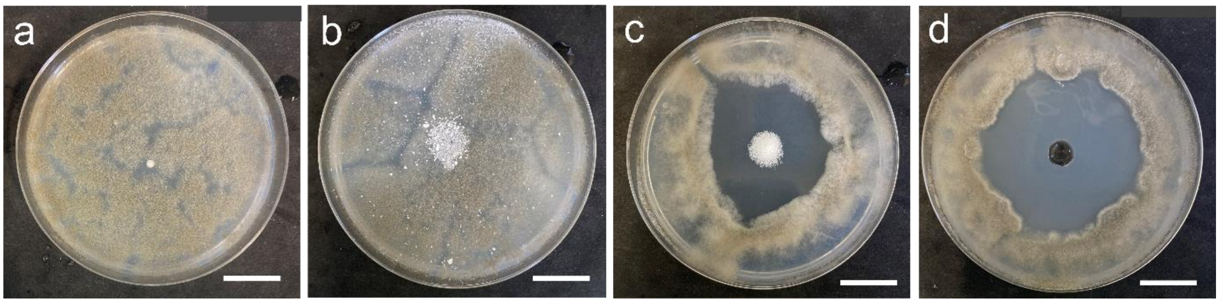

Figure S9: Digital images of B. cinerea growth on PDA agar (t=7 days) with: (a) Control (without added fatty acid). (b) Stearic acid. (c) Sorbic acid. (d) Caprylic acid. Scalebar is 2 cm.

The results shown in Figure S9 reveal that pure stearic acid powder exhibits no inhibition effect on *B. cinerea* growth compared to the control agar sample without fatty acids (Figure S9 b vs. a). In contrast, both pure sorbic and caprylic acids inhibit *B. cinerea* growth, with inhibition rates of 52 ± 10% and 64 ± 8%, respectively. The enhanced inhibition effect of MCFAs in this experiment may be attributed to their higher diffusion rate in agar compared to the long-chain stearic acid molecule. While this method cannot definitively exclude stearic acid's inhibition of *B. cinerea* growth, it clearly highlights the superior intrinsic antimicrobial effects of caprylic and sorbic acids relative to stearic acid in their native forms.

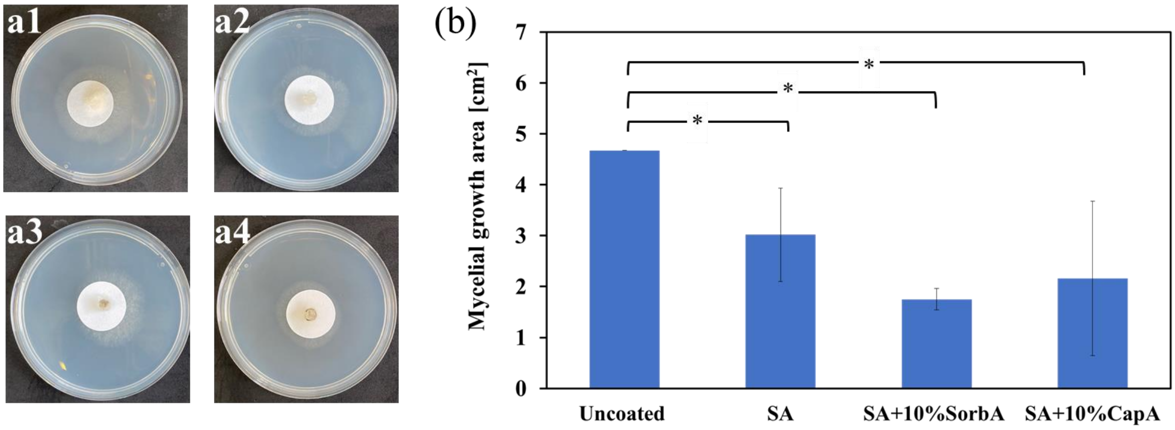

Figure S10: Antifungal activity of 8-day-old fatty acid superhydrophobic coatings. (a) Digital images showing the mycelial growth of B. cinerea on coated papers: (a1) Uncoated paper (control), (a2) Stearic acid coating, (a3) Stearic with 10% sorbic acid coating, (a4) Stearic with 10% caprylic acid coating. (b) Measured mycelial growth area values above the filter papers for coated and uncoated paper. (*) indicates significant difference (p-value ≤ 0.05) according to a two-tail test.

Figure S10 shows the *B. cinerea* mycelial growth of 8-day-old coatings. In this experiment, the coatings were stored under ambient conditions for 8 days and then exposed to *B. cinerea* mycelial agar plug, as described in the experimental section. Antifungal activity was assessed based on visual observation of mycelial growth on coated filter papers after 72 h.

References

(1) Webb, H. K.; Truong, V. K.; Hasan, J.; Fluke, C.; Crawford, R. J.; Ivanova, E. P. Roughness Parameters for Standard Description of Surface Nanoarchitecture. *Scanning* **2012**, *34* (4), 257–263. https://doi.org/10.1002/sca.21002.

(2) Kelton, K. F. *Crystal Nucleation in Liquids and Glasses*; 1991; Vol. 45. https://doi.org/10.1016/S0081-1947(08)60144-7.
